# Curcumin and Its New Derivatives: Correlation between Cytotoxicity against Breast Cancer Cell Lines, Degradation of PTP1B Phosphatase and ROS Generation

**DOI:** 10.3390/ijms221910368

**Published:** 2021-09-26

**Authors:** Tomasz Kostrzewa, Karol Wołosewicz, Marek Jamrozik, Joanna Drzeżdżon, Julia Siemińska, Dagmara Jacewicz, Magdalena Górska-Ponikowska, Marcin Kołaczkowski, Ryszard Łaźny, Alicja Kuban-Jankowska

**Affiliations:** 1 Department of Medical Chemistry, Faculty of Medicine, Medical University of Gdansk, 80-211 Gdansk, Poland; magdalena.gorska-ponikowska@gumed.edu.pl; 2 Faculty of Chemistry, University of Bialystok, Ciolkowskiego 1K, 15-245 Bialystok, Poland; k.wolosewicz@uwb.edu.pl (K.W.); julia.sieminska@umb.edu.pl (J.S.); lazny@uwb.edu.pl (R.Ł.); 3 Department of Medicinal Chemistry, Faculty of Pharmacy, Jagiellonian University Medical College, 30-688 Krakow, Poland; marek.jamrozik@doctoral.uj.edu.pl (M.J.); marcin.kolaczkowski@uj.edu.pl (M.K.); 4 Department of Environmental Technology, Faculty of Chemistry, University of Gdansk, Wita Stwosza 63, 80-308 Gdansk, Poland; joanna.drzezdzon@ug.edu.pl (J.D.); dagmara.jacewicz@ug.edu.pl (D.J.); 5 Metabolomics Laboratory, Clinical Research Center, Medical University of Bialystok, 15-276 Bialystok, Poland; 6 The Euro-Mediterranean Institute of Science and Technology, 90139 Palermo, Italy; 7 Institute of Biomaterials and Biomolecular Systems, Department of Biophysics, University of Stuttgart, 70174 Stuttgart, Germany

**Keywords:** curcumin derivatives, PTP1B phosphatase, breast cancer, ROS generation

## Abstract

Breast cancer is the most common cancer of women—it affects more than 2 million women worldwide. PTP1B phosphatase can be one of the possible targets for new drugs in breast cancer therapy. In this paper, we present new curcumin derivatives featuring a 4-piperidone ring as PTP1B inhibitors and ROS inducers. We performed cytotoxicity analysis for twelve curcumin derivatives against breast cancer MCF-7 and MDA-MB-231 cell lines and the human keratinocyte HaCaT cell line. Furthermore, because curcumin is a known antioxidant, we assessed antioxidant effects in its derivatives. For the most potent cytotoxic compounds, we determined intracellular ROS and PTP1B phosphatase levels. Moreover, for curcumin and its derivatives, we performed real-time microscopy to observe the photosensitizing effect. Finally, computational analysis was performed for the curcumin derivatives with an inhibitory effect against PTP1B phosphatase to assess the potential binding mode of new inhibitors within the allosteric site of the enzyme. We observed that two tested compounds are better anticancer agents than curcumin. Moreover, we suggest that blocking the -OH group in phenolic compounds causes an increase in the cytotoxicity effect, even at a low concentration. Furthermore, due to this modification, a higher level of ROS is induced, which correlates with a lower level of PTP1B.

## 1. Introduction

**Figure 1:**
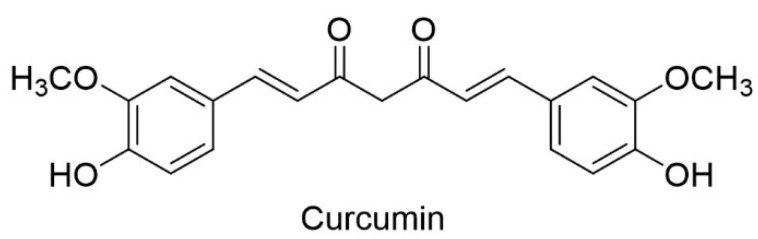
Structure of curcumin (ketone tautomer).

The prevalence of various types of cancer leads scientists to look for new active substances that can serve as medicines. Although there are many anti-cancer drugs being revealed, there is still a significant number of limitations that prevent effective therapy, such as multi-drug resistance, unselective targeting of cancer and normal cells, poor outcomes, and recurrence of cancer (relapse of cancer). Among the various groups of potential drug candidates, low molecular weight organic compounds are a major group and many of them are derived from biomolecules of natural origin. One of the prospective substances, due to a wide range of activity in vivo tests and low toxicity, is curcumin [(1,7-bis(4-hydroxy-3-methoxyphenyl)-1,6-heptadiene-3,5-dione)] (Figure 1) [1,2].

This phenolic compound is a constituent of the natural ingredient of the rhizome of *Curcuma longa* and is widely used in traditional eastern medicine. As a food additive, it can be safely consumed in huge doses—up to 12 g in a single dose [3]. Curcumin, in the tests conducted, shows antioxidant [4], anti-inflammation [5], anti-fungal [6], anti-cancer [7], and many other biological activities [8,9]. Although curcumin has many promising properties, it has poor bioavailability and a weak pharmacokinetic profile. The reasons for this are poor solubility in aqueous gastrointestinal fluids, week absorption in the gastrointestinal tract, and instability at physiological pH—at a pH of 7.2–8.0, the half-life of curcumin is only 1 to 9 min. The presence of proteins increases stability, but after 1h about 20% of curcumin is still degraded in human blood [10]. The main obstacles in the application of curcumin as an active pharmaceutical ingredient are its fast metabolism and elimination from the human body, poor water solubility, and chemical instability [11].

Many biological activities and negligible negative impacts on the human body (turmeric, the constituent of which is curcumin, has been used in Indian cuisine for centuries) [12] prompts scientists to look for a way to improve the bioavailability of curcumin [13] or to develop its derivatives with increased bioavailability [8]. One of the strategies to improve the bioavailability of curcumin analogues is the incorporation of a nitrogen atom in the molecule. Such a molecule will have a secondary interaction site—a hydrogen bond acceptor. This modification may increase the antiproliferative properties of the tested compounds [14]. The effect of incorporating a nitrogen atom in the structure of curcumin analogues with a 4-piperidone cyclic core was studied [14,15,16], and some of the tested compounds demonstrated very good antiproliferative activities [17]. For this reason, we decided to incorporate the heterocyclic structure of 4-piperidone into a curcumin molecule. Unfortunately, the majority of studied analogues with a 4-piperidone ring were monocarbonyl analogues of curcumin (MACs) [8,17,18]. The lack of a 1,3-diketone moiety characteristic for curcumin in those compounds may significantly affect their biological activity. It was reported that the enol form of 1,3-diketone is responsible for some curcumin activity. The curcumin analogues that cannot take the enol form (have a fluorine atom instead of hydrogen in the methylene position between carbonyl groups) showed significantly lower activity in the anti-androgen activity in LNCaP and PC-3 prostate cancer cells tests compared to the enolizing (non-fluorinated) compounds [19]. Unfortunately, curcumin analogues with a 4-piperidone structure fused with the 1,3-diketone moiety are, to the best of our knowledge, unknown. For these reasons, we decided to investigate 1,3-diketone curcumin analogues with a 4-piperidone ring in our research.

Protein tyrosine phosphatases (PTPs) regulate the tyrosine phosphorylation process, which is responsible for the control of cell adhesion and migration. The human genome contains more than one hundred different genes of protein tyrosine phosphatases (just as protein tyrosine kinase genes) coding for approximately five types of phosphatases. A large number of existing various protein tyrosine phosphatases result from the fact that PTP genes are able to produce different products through a process called alternative splicing, which in turn may undergo post-translational modifications [20]. Phosphorylation and dephosphorylation of the tyrosine residues of proteins is an evolutionarily preserved mechanism of signal transduction in eukaryotic cells that is of fundamental importance in the control of cell physiology, including proliferation, differentiation, migration, and tumorigenesis. Reversible tyrosine phosphorylation of proteins is regulated by a balance between the antagonistic action of protein tyrosine phosphatases and tyrosine kinases [21]. Their balanced and opposing action is crucial for the maintenance of homeostasis, and any interference can contribute to the development of the diseases and the process of carcinogenesis. Abnormal tyrosine phosphorylation may lead to cancer characterized by abnormal growth and metastatic potential. There is evidence that some of the PTPs can stimulate the process of tumorigenesis because many protein tyrosine phosphatases in human organisms are responsibilities for regulating other proteins and their malfunction may cause activity of growth factors and may promote tumor formation [22,23]. The role of protein tyrosine phosphatases in the formation and development of tumors was presented during the implementation of a number of scientific research studies. The participation of PTPs in the development of glioma, colorectal cancer, lung cancer, breast cancer, and multiple myeloma has been proven [24].

Regulatory mechanisms of PTP1B play a significant role in the proper functioning of the immune system, and altered expression of the gene encoding this enzyme may be one of the causes of some types of cancer, autoimmune diseases, metabolic diseases, or viral infections. Due to the above, modulation of PTP1B phosphatase activity with inhibitors may play an important role in modern pharmacotherapy. Therefore, we also tested the new curcumin derivatives for inhibitory activity against PTP1B phosphatase.

## 2. Results

### 2.1. Synthesis

**Figure 2:**
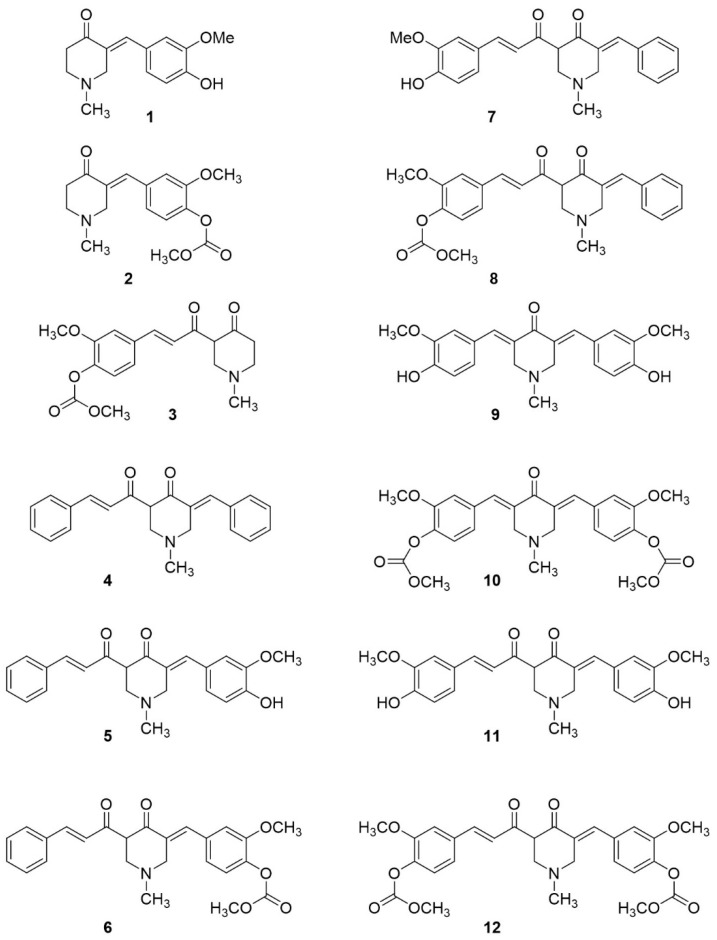
Structures of the obtained curcumin analogues. The first step of the synthesis was the aldol reaction of 4-piperidone and selected aldehydes (benzaldehyde or 3-methoxy-4-methoxycarbonyloxy-benzaldehyde) promoted by lithium diisopropylamide (LDA) in anhydrous tetrahydrofuran (THF). The obtained aldols were dehydrated (elimination of water) in acidic conditions to obtain monosubstituated benzylidene piperidones. We chose a complicated procedure using LDA in anhydrous conditions to avoid obtaining only the thermodynamically stable product of attachment of two aldehyde molecules, the formation of which was observed under both acidic and basic conditions [16,26]. In the synthesis of compound **2**, the hydroxyl group of aldol was acetylated (catalyzed by DMAP: 4-(dimethylamino)pyridine) before acid-catalyzed elimination. This transformation allows one to perform the elimination reaction under milder conditions (without heating) and increase the yield of obtained product [25]. Unfortunately, despite those preventive measures, in addition to the desired product **2** (29% yield), 2,4-disubstituated piperidone **10** (14% yield) was obtained (Scheme 1).

Synthesis of most of the investigated compounds (Figure 2) was conducted according to a procedure reported before [25]. Only compound **9,** which was synthesized by the direct reaction of 4-piperidone and vanillin under acidic conditions [26], and compound **1** were commercially available.

The activity of both products (compound **2** and compound **10**) was examined in our research. The configuration of the 2-benzylidenepiperidones obtained has been confirmed as *E*, based on chemical shifts in the ^1^H-NMR spectra (the olefinic H singlet was observed at 7.50 ppm **2**, 7.74 ppm **10,** and 7.53 **9**). The typical chemical shifts in the ^1^H-NMR spectra for the *Z* isomers are in the range of 6.0–6.5 ppm [27].

**Scheme 2:**
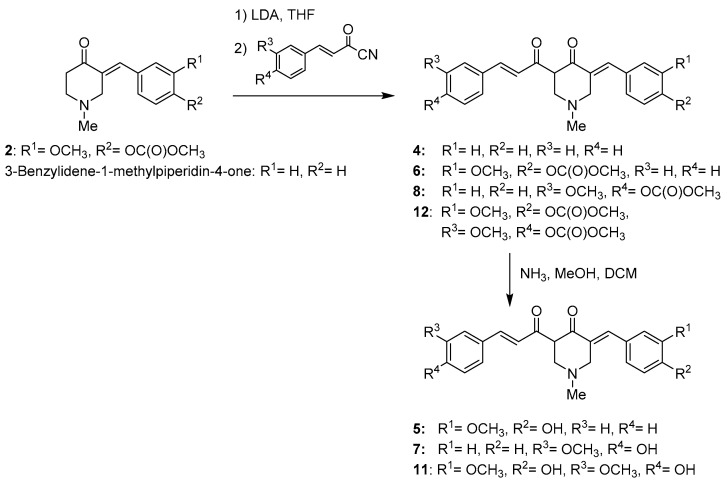
Synthesis of dicarbonyl curcumin analogues.

In the next step, lithium enolates generated in the reaction of the ketones with LDA were acylated with acyl cyanides affording compounds **4**, **6**, **8**, and **12** (from α-benzylidenepiperidones) or compound **3** (from 4-piperydone, Scheme 2). Deprotection of the hydroxyl group was affected under alkaline conditions (10% ammonia solution in water) affording products **5** and **7** in moderate yields (62% and 48%). Only compound **11** was obtained with a lower yield (35%) due to difficulty in purification of the product— chromatography repeated several times resulted in an impure product, which had to be purified by recrystallization with dichloromethane (DCM). The constant *J* values and the chemical shift of the double bonds of the crotonyl moieties in the ^1^H-NMR spectra were similar to the acyl cyanides, which confirmed that the product has an unchanged *E* configuration. The chemical shift above 16 ppm of the hydrogen in the methylene position between carbonyl groups in the ^1^H-NMR spectra proved that the 1,3-dicarbonyl moiety was predominantly in the enol form. That fact has been confirmed in the literature by X-ray of a similar compound [25]. However, in an analogy to the curcumin molecule, which is usually drawn in the keto form, we decided to show the curcumin analogues with a 1,3-diketone moiety in the ketone form in this paper.

### 2.2. Low Concentration of Synthesized Derivatives of Curcumin Showed Antioxidant Effect

All tested compounds are characterized by high reactivity towards the superoxide radical anion. Curcumin and its derivatives react with the superoxide anion to form a product with maximum absorption at 560 nm. On the other hand, for the purpose of the studies described in this report, such low concentrations of compounds were selected so that the possibility of product formation did not interfere with the absorbance measurements. After the antioxidants reacted with the superoxide anion, part of the superoxide anion radicals in the tested sample remained and reacted with nitro blue tetrazolium (NBT) to form formazan. The values of percent superoxide anion scavenging by curcumin and its derivatives are presented in Table 1.

The results show that compound **12** and compound **8** are better antioxidants than curcumin, which is known as a good antioxidant. The chemical compound **9** is also worth attention because at a low concentration showed relatively high antioxidant activity.

### 2.3. Curcumin and Its Derivatives Show Cytotoxicity Effect against Breast Cancer Cell Line

We assessed the cytotoxicity and calculated IC_50_ values for curcumin and its new derivatives against breast cancer MCF-7 and MDA-MB-231 cell lines and the human keratinocyte HaCaT cell line (as a non-cancerous control). Cells were treated with serial concentrations (7.81 to 100 µM) of each compound, and then the cells were incubated for 24 h. Based on the obtained results, we determined the IC_50_ using a nonlinear log (inh) vs. normalized response–variable slope (Table 2) for compounds, which showed 50% cytotoxicity below the 100 µM concentration level.

**Figure 3:**
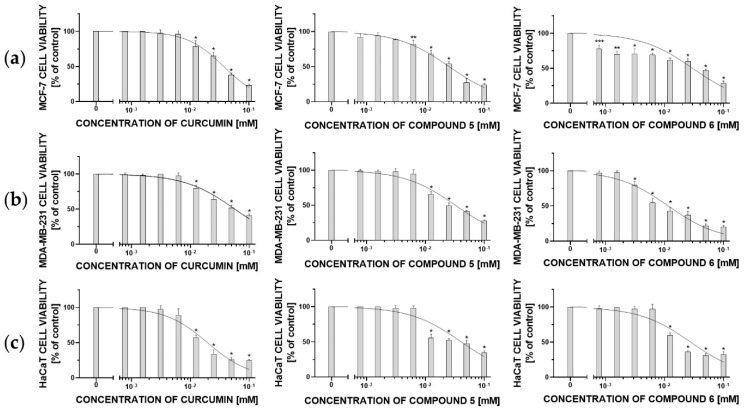
The cellular viability of the (**a**) MCF-7 cell line, (**b**) MDA-MB-231 cell line, and (**c**) HaCat cell line, after 24 h incubation with different concentrations of curcumin, compound **5**, and compound **6**. The cellular viability was measured by MTT Cell Viability assay. The results were presented as a percentage of control (mean ± SD, *n* = 3, * *p* < 0.0001; ** *p* < 0.01; *** *p* < 0.1).

We observed that compounds **5** and **6** showed a better cytotoxic effect against both types of breast cancer cell lines in comparison to all tested compounds. Against the MCF-7 cell line, the cytotoxic agents better than curcumin are compounds **3**, **10,** and **11**. It is worth noting that compounds **5** and **6** showed a higher selectivity index than other compounds, both against MCF-7 and MDA-MB-231 cell lines. Based on the ratio of the IC_50_ values determined for non-tumor-transformed cells to breast cancer cells, it can be observed that compound **5** showed cytotoxicity 1.63 times higher against MCF-7 cells compared to HaCaT cells and 1.29 times higher against MDA-MB-231 cells compared to HaCaT cells. On the other hand, its analogue (compound **6**) showed similar cytotoxicity in the concentration range of 12.5–100 µM for MCF-7 and HaCat cells; however, it showed 2.61 times higher cytotoxicity for MDA-MB-231 cells than for HaCat cells. Moreover, it is worth noting that both compounds **5** and **6** have substituents on the aromatic ring proximal to the piperazine ring. The difference between these compounds was the protection of the -OH group at position 4 in the aromatic ring (compound **6**) compared to the free -OH group (compound **5**). Protection of the -OH group increased cytotoxicity in the low concentration range of 7.81–12.5 against the MCF-7 cell line (reduce viability to 78–62%) and in the concentration range of 3.125–6.25 µM against MDA-MB-231 (reduced viability to 79–55%) cells (Figure 3).

### 2.4. Generation of Intracellular ROS Level Corresponds to Degradation of PTP1B

**Figure 4:**
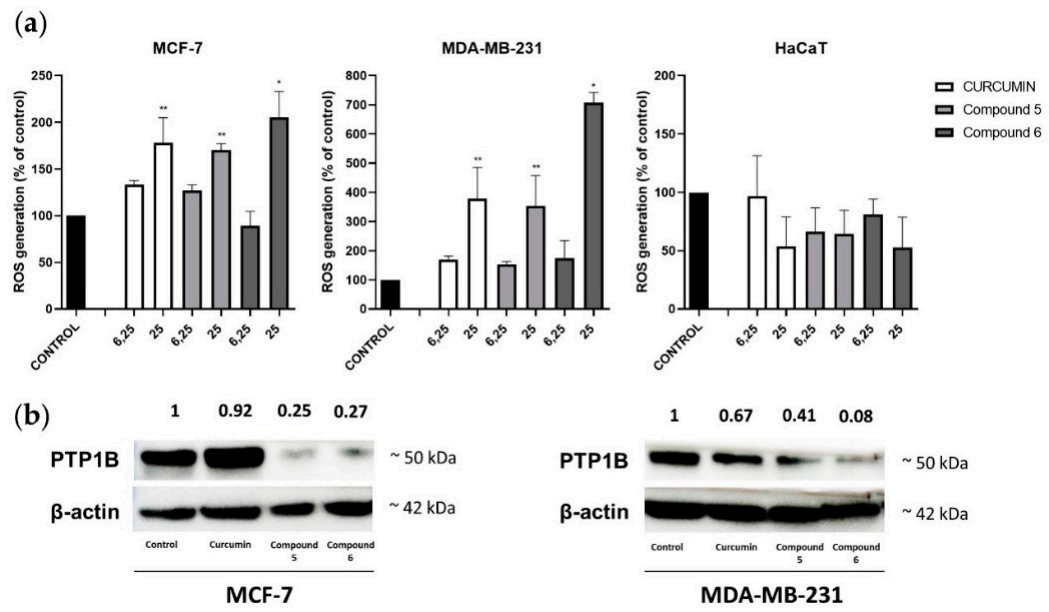
(**a**) The levels of ROS induced by curcumin, compound **5**, and compound **6** in concentrations of 6.25 and 25 µM in MCF-7, MDA-MB-231, and HaCaT cell lines after 24 h of incubation. The results were presented as a percentage of control (mean ± SD, *n* = 3, * *p* < 0.0001; ** *p* < 0.01); (**b**) Western blot of PTP1B expression in cancer cells.

Based on the cytotoxicity results obtained, for analysis of intracellular reactive oxygen species (ROS) generation in MCF-7, MDA-MB-231, and HaCat cell lines, we chose curcumin and its two derivatives—compound **5** and compound **6**. We observed that curcumin, compound **5**, and compound **6**, in a concentration of 25 µM, triggers the generation of intracellular ROS levels in the breast cancer cell line, whereas they did not generate ROS in a non-tumor-transformed HaCaT cell line. The strongest ROS generation was induced by compound **6** in a concentration of 25 µM, both in MCF-7 (205.49 ± 27.65%) and MDA-MB-231 (707.29 ± 34.81%) cell lines. To correlate the effect of intracellular ROS generation with the degradation of PTP1B phosphatase, we performed Western Blot analysis for curcumin, compound **5**, and compound **6** against breast cancer cell lines. As we noted, compound **6** was responsible for the strongest degradation of PTP1B phosphatase in MDA-MB-231 cell lines, while in the MCF-7 cell line, compound **5** was slightly stronger. On the other hand, in two tested cell lines, curcumin had the lowest effect on PTP1B levels (Figure 4).

### 2.5. Curcumin and Compound **5** Show a Photosensitizing Effect

**Figure 5:**
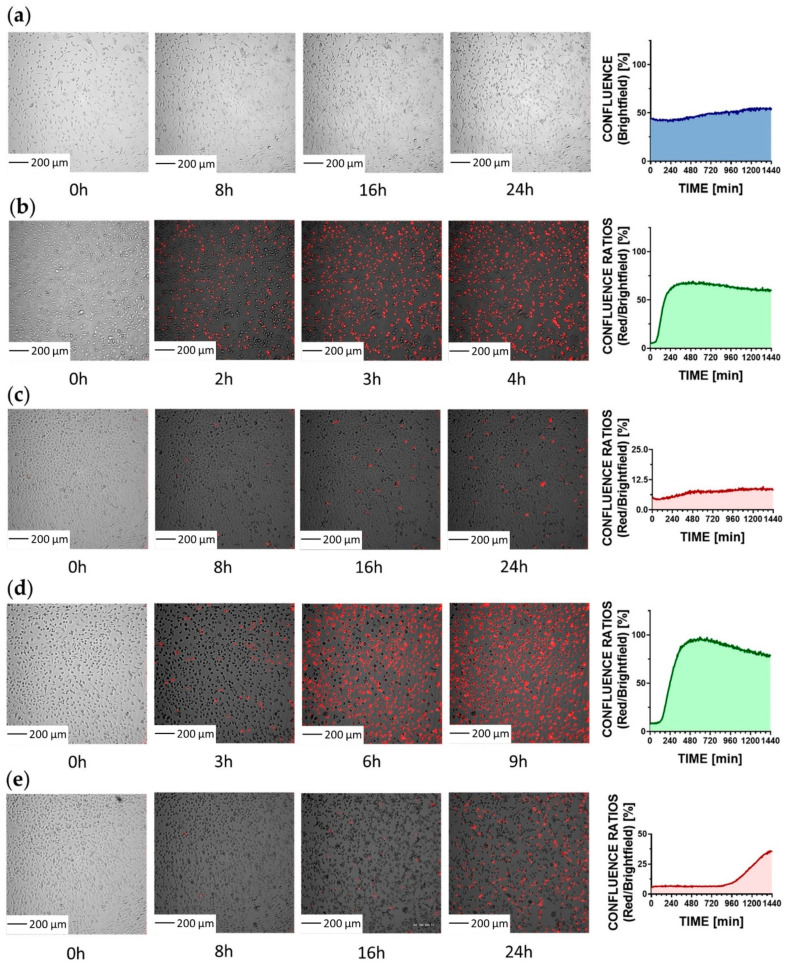
Real-time fluorescence microscopy. Snapshots are presented with the red channel enabled to visualize late apoptotic/necrotic cells. (**a**) Control cells snapshots—cells were treated with propidium iodide (PI) and registered with brightfield, green, and red channels enabled. The graph shows the confluence of cells. (**b**) MDA-MB-231 cells treated with PI and 25 µM curcumin and registered with brightfield, green, and red channels at the same time. The graph shows confluence ratios for red/brightfield channels; (**c**) MDA-MB-231 treated with PI and 25 µM curcumin and registered with the brightfield and red channels enabled. The graph shows confluence ratios for red/brightfield channels; (**d**) MDA-MB-231 cells treated with PI and 25 µM compound **5** and registered brightfield, green, and red channels enabled at the same time. The graph shows confluence ratios for red/brightfield channels. (**e**) MDA-MB-231 treated with PI and 25 µM compound **5** and registered with the brightfield and red channels enabled. The graph shows confluence ratios for red/brightfield.

The absorption spectrum range for curcumin is between 300 and 500 nm with a maximum absorption at 435 nm. Many scientific articles indicate that curcumin can be used as a photosensitizer in photodynamic therapy, both against cancer and bacterial cells [32,33,34]. In our study, we observed the viability of MDA-MB-231 cells treated with curcumin and compound **5** in concentrations of 25 µM using a real-time fluorescence microscope. Snapshots were taken every 5 min for 24 h, using brightfield, green, and red channels in parallel, or brightfield and red channels in parallel. As we noticed, with simultaneous irradiation with the green and red channels (Figure 5b,d), the cytotoxicity of both compounds was higher than when only the red channel was used (Figure 5c,e). We also found that compound **5** showed higher cytotoxicity after irradiation with green light than curcumin.

### 2.6. Curcumin and Compound **6** Shows Inhibitory Effect against Protein Tyrosine Phosphatase PTP1B

**Figure 6:**
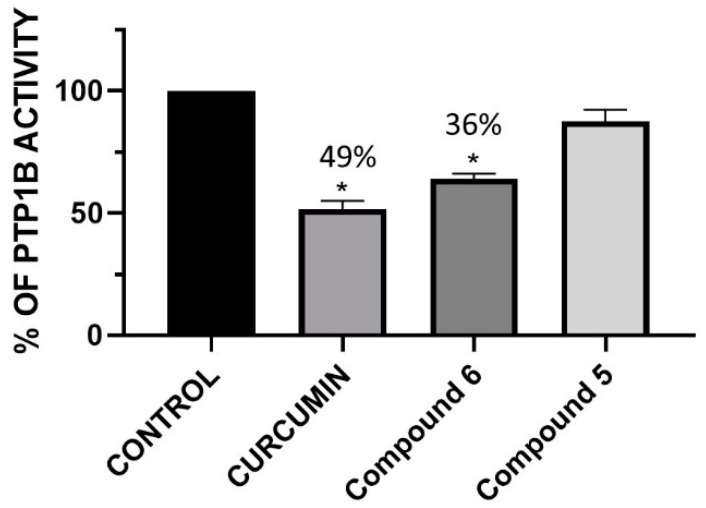
Enzymatic activity of PTP1B after 30 min of treatment with different concentrations of curcumin, compound **6**, and compound **5**. The results were presented as a percentage of control as means ± SD (*n* = 3, * *p* < 0.0001).

Due to the previously determined inhibition of PTP1B by curcumin [35], for the obtained derivatives, we conducted a study of the inhibitory potential against protein tyrosine phosphatase PTP1B. Except compound **6**, all the derivatives obtained at a concentration of 100 µM showed no inhibitory potential towards PTP1B. Compound **6** reduced PTP1B ability to dephosphorylate the substrate by 36%, while curcumin showed a 51% inhibitory effect (Figure 6). Therefore, we performed a molecular docking simulation for compound **6** on the PTP1B crystal.

**Figure 7:**
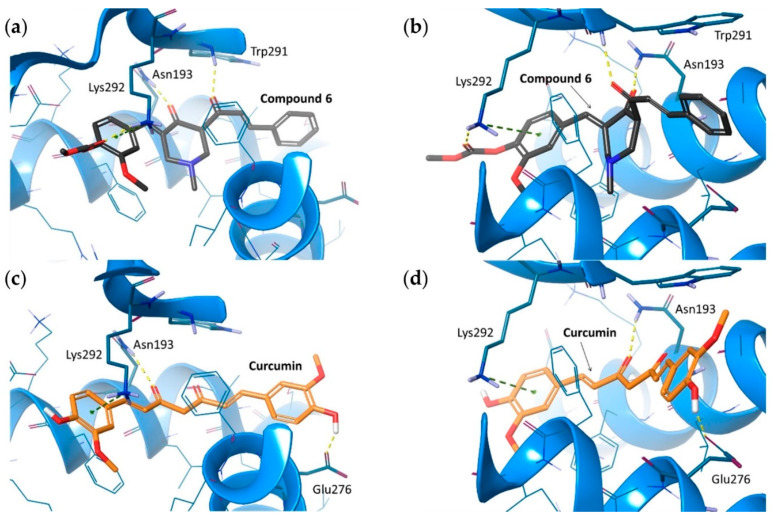
Molecular interactions formed between PTP1B (code 1T48) amino acid residues and its docked allosteric inhibitors: Compound **6** (**a**,**b**) and curcumin (**c**,**d**). Amino acid residues within 5 Å displayed as thin, blue sticks; amino acid residues engaged in ligand binding throughout H-bonds (dotted yellow lines) and π-cation interaction (dotted green lines) displayed as bold, blue sticks.

For the purpose of this research, we selected the PTP1B crystal structure 1T48, which was originally co-crystallized together with co BB3 ligand (benzbromarone derivative), bound in the allosteric site of the enzyme. First, we performed Induced-Fit docking (IFD) to evaluate the putative binding mode of compound **6**. As a result, we obtained a complex with several molecular interactions between compound **6** and selected PTP1B amino acid residues (Figure 7a,b). The carbonyl group from the 1-methylpiperidin-4-one moiety of compound **6** formed an H-bond with the side chain of Asn193. The same interaction was observed between BB3 and PTP1B in the 1T48 crystal structure. Additionally, the second carbonyl group from compound **6** formed an H-bond with the main chain of Trp291. We also observed H-bond and π-cation interactions between Lys292 and the methyl phenyl carbonate fragment of compound **6**. Those two amino acid residues were also involved in the formation of interactions in 1T48, but their types were different (π-π stacking and salt bridge, respectively). However, the presence of the same residues taking part in the protein–ligand interaction of both the aforementioned ligands suggests an increased likelihood of high affinity of compound **6** to the allosteric site of PTP1B.

**Figure 8:**
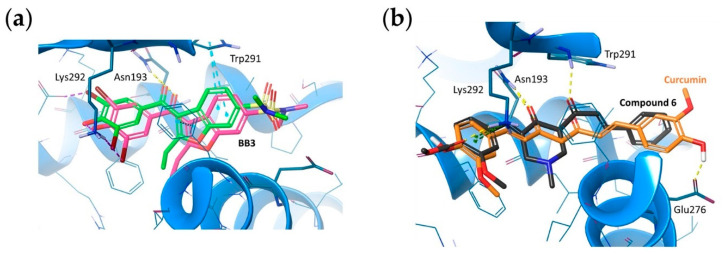
(**a**) Spatial orientation of BB3 within the allosteric site of PtP1B in the original crystal structure (PDB ID: 1T48; green ligand) and docked to the obtained, optimized model (pink ligand). RMSD obtained for BB3 redocking: 1.66 Å. CBR1 backbone displayed as blue ribbon. Amino acid residues engaged in ligand binding throughout H-bonds (dotted yellow lines), π-interactions (dotted blue lines), and halogen bonds (dotted purple lines) are displayed as sticks. (**b**) The predicted binding mode comparison of compound **6** (black) and curcumin (orange) within the allosteric site of PTP1B. Amino acid residues within 5 Å displayed as thin, blue sticks; amino acid residues engaged in ligand binding throughout H-bonds (dotted yellow lines) and π-cation interaction (dotted green lines) displayed as bold, blue sticks.

After basic evaluation of the obtained PTP1B-compound **6** complex, we redocked BB3. RMSD of redocked BB3 vs. the one from the crystal structure was 1.66 Å^2^, which proves the good quality of the model we used (Figure 8a).

Based on the obtained results, we used the described model to dock another allosteric PTP1B inhibitor—curcumin. As a result, we obtained a binding mode similar to the one observed for compound **6** (Figure 7c,d). Molecular interactions with Asn193 (H-bond) and Lys292 (π-cation) were observed, with an additional H-bond between one of curcumin’s phenolic groups and Glu276. The superposition of compound **6** and curcumin indicated a similar position of potential pharmacophore features (two H-bond acceptors and two aromatic rings) of both ligands within the PTP1B allosteric site (Figure 8b).

In the described docking simulations, we did not observe any interaction with Phe280 that was detected in 1T48 but based on both in silico and in vitro results we assume that this interaction is not required for effective allosteric inhibition of PTP1B.

## 3. Discussion

The biggest threat and the main cause of cancer-related mortality is metastatic potential. After chemotherapy or radiotherapy, it is important to prevent the formation of a secondary tumor. The molecular mechanisms underlying metastasis are key to developing new strategies for the prevention and treatment of cancer.

PTP1B protein phosphatase is overexpressed in breast cancer cells, where it induces tumor growth by promoting the overexpression of growth factors and cytokines [36]. Researchers indicate that PTP may be a potential therapeutic target in the prevention and treatment of breast cancer [37]. It has not yet been established whether it is possible to treat advanced stages of human epidermal growth factor receptor 2 (HER2, Erb2, HER2/neu)-positive breast cancer by inhibiting PTP1B. However, it was found that the deletion of PTP1B in mice reduces the risk or at least delays HER2/Neu-induced carcinogenesis, and overexpression of PTP1B in the mammary gland leads to the spontaneous development of breast cancer. PTP1B is involved in the control of Erb2-induced tumorigenesis of breast cancer by the attenuation of mitogen-activated protein kinases (MAP, MAPK) and Akt pathways [38].

In studies comparing neoplastic cells to healthy ones, PTP1B overexpression in tumor cells is significant. Researchers point to a correlation of tumor size and lymph node metastasis in patients who have higher levels of PTP1B [39]. PTP1B promotes invasion and metastasis by regulating the PTEN-AKT/pAKT pathway and promoting the expression of metalloproteinase 2 (MMP2) and MMP7, which is associated with lymph node metastasis. In addition, it was noted that the impact of PTP1B on phosphatase and the tensin homolog was the deletion of chromosome ten (PTEN), accompanied with an abatement of AKT phosphorylation [40]. Moreover, PTP1B dephosphorylates the signal transducer and activator of transcription 3 (STAT3), which in turn increases C-C motif chemokine ligand 5 (CCL5) expression responsible for the invasion of cancer cells [39].

Many studies have shown that PTP1B can act as an activator of the Src kinase, which may result in increasing tumorigenicity and promoting tumor progression. Non-receptor tyrosine kinase Src is deregulated in multiple tumor types. The crucial role of Src kinases in tumor development involves the effect of Src on proliferation, survival, adhesion, migration, invasion, and metastasis. Src kinase activity has been reported to be highly elevated in many human cancer cell lines, e.g., breast cancer, lung cancer, and colon cancer cells [41,42,43,44,45]. Moreover, studies of several human breast cancer cell lines with increased Src kinase activity have shown higher levels of the PTP1B protein relative to healthy breast epithelial cells. There are two main Src phosphorylation sites: The Cys419 autophosphorylation site and the Cys530 C-terminal phosphorylation site. PTP1B dephosphorylates Src kinase and, through activation, controls its pro-oncogenic effect. Acting together, they contribute to the development and progression of breast cancer [46].

PTPs play an important part in cell signaling. Moreover, they can be indicative of the reactive oxygen species (ROS) level because reversible oxidation is the main mechanism of control of PTPs activity. A ROS level higher than normal is one of the reasons for cancer development and progression [47]. Several studies suggest that the production of reactive oxygen species associated with oxidative stress may lead to the inactivation of protein tyrosine phosphatases. In addition, reversible oxidation of catalytic cysteine is suggested to be a major regulatory mechanism of PTPs activity [48]. Generally, reversible oxidation of the thiol moiety to sulfenic acid is a biological process commonly used to control the functions of many proteins. These thiol groups containing proteins play an important role in cell signaling (e.g., PTP1B), binding transcription factors to DNA (e.g., Nrf-2, NF-κβ), and other regulatory processes [49]. Due to high vulnerability to oxidation and relatively easy activity measurement, PTPs may be used as extremely sensitive biosensors of oxidative stress levels. Thereupon, the role of PTPs in the genesis of cancers may partly result from the increased level of ROS in tumor cells compared to healthy cells, which leads to impaired regulation of PTPs by oxidation [50]. Furthermore, overexpression of the protein Nox1 producing reactive oxygen species can cause cellular transformation and tumorigenesis, confirming the participation of ROS in cancer development [51].

In this study, we examined the cytotoxicity effect on breast cancer cells in correlation to different places of substitution on aromatic rings relative to the piperazine ring in curcumin derivatives. The compounds synthesized by our group are characterized by the free -OH group or blocked by carbonic acid ester. The main reason for the study of the activity of derivatives with a protected hydroxyl group was to reduce the metabolism of the proposed substances, which is described as one of the main (next to poor water solubility) factors limiting bioavailability and, as a result, the use of curcumin in medicine. Literature data indicate that curcumin sulfates(VI) and glucuronates linked to the curcumin phenol group are the major metabolites of curcumin [52]. Blocking the phenol group in the tested derivatives caused them to slow down their metabolism, enabling the use of a smaller dose of the substance to induce a cytotoxic effect. We observed that compounds **5** and **6** were better cytotoxicity agents than other derivatives. It is worth emphasizing that these compounds are characterized by substitution in the aromatic ring proximal to the piperazine ring and that the difference between these compounds was the protection of the -OH group at position 4 of the aromatic ring. This protection caused compound **6** to show high antioxidant effectiveness in a low concentration (scavenging 6% in concentration 0.05 µM), higher cytotoxicity potential against triple-negative breast cancer, and the generation of a higher level of ROS. Moreover, the higher level of ROS correlated with the lower level of PTP1B in MDA-MB-231 cell lysates. On the other hand, in silico studies showed that compound **6** could be an allosteric inhibitor of PTP1B. Curcumin is indicated as a pan-assay interference compound (PAINS) [53], which means it gives false positive results against numerous proteins. In our research, the synthesized derivatives did not show this effect. Furthermore, in the recombinant PTP1B assay, we observed that compound **6** caused an inhibitory effect in a concentration of 100 µM.

## 4. Materials and Methods

### 4.1. Chemicals and Reagents

*N*-Methyl-4-piperidone, Benzaldehyde, Diisopropylamine, *n*-Butyllithium (2.5 M solution in hexanes), 4-(Dimethylamino)pyridine (DMAP), and Sodium bicarbonate were purchased from Merck. Acetic acid, Dichloromethane, Tetrahydrofuran, Ethanol, Ethyl acetate, Citric acid, Sodium Sulfate(VI), and Acetic anhydride were purchased from POCH, Gliwice, Poland. Vanilin was purchased from Acros, Waltham, Massachusetts, USA. Dimethyl-sulfoxide (DMSO), Potassium superoxide, 18-crown-6-ether, Nitro blue tetrazolium chloride (NBT), Curcumin, Fetal bovine serum (FBS), 3-(4,5-dimethylthiazol-2-yl)-2,5-diphenyltetrazolium bromide (MTT), recombinant PTP1B phosphatase, *para*-nitrophenyl phosphate (*p*NPP), and 2′,7′-dichlorofluorescein were purchased from Sigma-Aldrich, Saint Louis, Missouri, USA. Dulbecco’s Modified Eagle’s Medium (DMEM) and Phosphate-buffered saline (PBS) were purchased from PAN-biotech, Aidenbach, Germany. Lastly, 4–20% MP TGX Tain-Free Gel 10W was purchased from Bio-Rad Laboratories, Hercules, CA, USA and PTP1B antibodies were purchased from Cell Signaling, Danvers, MA, USA.

### 4.2. Synthesis

#### 4.2.1. General Information

The ^1^H and ^13^C NMR spectra were recorded on a Bruker AVANCE II 400 spectrometer (Bruker, Fallanden, Switzerland) at 400 MHz for ^1^H and 100 MHz for ^13^C NMR spectra. The chemical shifts are reported relative to tetramethylsilane (TMS) and were referenced to the CDCl_3_ signals at δ = 7.26 for ^1^H and δ = 77.0 for ^13^C spectra. IR spectra were recorded on the Nicolet 6700 (Thermo Scientific, Waltham, Massachusetts, USA); the Attenuated Total Reflectance (ATR) method was used. The EZ-Melt MPA120 apparatus (Stanford Research Systems, Sunnyvale, California, USA) was used to measure melting points. An LC-MS system consisting of 1290 Infinity UHPLC coupled to a 6545 Q-TOF-MS detector (Agilent, Santa Clara, CA, USA) or Micromass LCT 298 TOF (Waters, Milford, MA, USA) was used to record high-resolution mass spectra (accurate mass analysis). The analyses were carried out in a positive ion mode. Air-sensitive reactions were carried out under argon in flame-dried Schlenk tubes. Dichloromethane (DCM) and diisopropylamine (DIPA) were distilled from CaH_2_. Tetrahydrofuran (THF) was dried by distillation from sodium benzophenone ketyl under argon. The commercially available reagents were used without purification. Flash Column Chromatography (FCC) was performed on Silica gel 230–400 mesh from Fluka. The reagents used in the synthesis were commercially available or were synthesized by methods from the literature (a reference to their synthesis is given).

#### 4.2.2. Synthesis of the Compounds

**(3*E*)-3-[(4-Hydroxy-3-methoxyphenyl)methylidene]-1-methylpiperidin-4-one** (**1**) is a commercially available compound (from Aurora Fine Chemicals).

(*E*)-2-Methoxy-4-((1-methyl-4-oxopiperidin-3-ylidene)methyl)phenyl methyl carbonate (**2**) and dimethyl (((1*E*,1′*E*)-(1-methyl-4-oxopiperidine-3,5-diylidene)bis(methaneylylidene))bis(2-methoxy-4,1-phenylene)) bis(carbonate) (**10**).

To a dry, argon-filled Schlenk tube, anhydrous THF (100 mL) and diisopropyl amine (6.21 mL, 44.0 mmol) were added. The solution was cooled to 0 °C and then *n*-butyllithium was added (2.5 M solution in hexanes, 17.6 mL, 44.0 mmol). After 30 min, *N*-methyl-4-piperidone (4.92 mL, 40.0 mmol) was added dropwise to the reaction. The reaction was stirred at 0 °C for 60 min and then cooled to −78 °C and stirred for an additional 15 min, then a solution of 3-methoxy-4-methoxycarbonyloxy-benzaldehyde (10.089 g, 48.0 mmol) [54] in dry THF 32.0 mL was added via cannula. After 30 min, the reaction was terminated by the addition of 20 mL of a saturated aqueous solution of NaHCO_3_, and after a minute. 10 mL of a 10% water solution of citric acid was added, to avoid hydrolysis of the carbamate. After heating to room temperature, 100 mL of water was added, and the mixture was extracted with dichloromethane (4 × 50 mL). Combined organic extracts were dried over Na_2_SO_4_ and concentrated under a vacuum. The crude product was dissolved in dry DCM (100 mL). Acetic anhydride (6.0 mL, 63.5 mmol) and 4-(dimethylamino)pyridine (0.195 g, 1.60 mmol) were added to the solution. The reaction was stirred for 2 h at room temperature, then acetic acid (99%, 8 mL) was added to cause the elimination of the formed ester. After 15 min, the mixture was pureed in 30 mL of cold water cooled to 0 °C, and the saturated aqueous solution of NaHCO_3_ was added to neutral pH. The formed mixture was extracted with DCM (4 × 50 mL). Combined extracts were dried over Na_2_SO_4_ and evaporated under a vacuum. The residue was purified by flash column chromatography (EtOH:DCM:AcOEt, 5:25:70) to afford the products **2** (3.569 g, 29%) as a white solid and **10** (2.822 g, 14%) as a pale yellow solid.

**(E)-2-Methoxy-4-((1-methyl-4-oxopiperidin-3-ylidene)methyl)phenyl methyl carbonate (2)**

Mp: 116.0–118.0 °C; ^1^H NMR (400 MHz, CDCl_3_): δ = 7.50 (s, 1H), 7.14 (d, *J* = 8.1 Hz, 1H), 6.94–6.87 (m, 2H), 3.90 (s, 3H), 3.84 (s, 3H), 3.62 (s, 2H), 2.80 (t, *J* = 6.2 Hz, 2H), 2.65 (t, *J* = 6.2 Hz, 2H), 2.42 (s, 3H) ppm; ^13^C NMR (100 MHz, CDCl_3_): δ = 197.44, 153.52, 150.96, 140.36, 134.86, 133.94, 133.27, 122.43, 122.30, 114.62, 57.46, 55.91, 55.54, 52.60, 46.03, 38.94 ppm; FTIR (ATR, ṽ): 2943, 1752, 1669, 1596, 1514, 1442, 1315 cm^−1^; HRMS (ESI): calcd. for: C_16_H_20_NO_5_ [M + H]^+^ 306.1336; found 306.13416.

**Dimethyl (((1E,1′E)-(1-methyl-4-oxopiperidine-3,5-diylidene)bis(methaneylylidene))bis(2-methoxy-4,1-phenylene)) bis(carbonate)** (**10**)

Mp: 154.0–157.0 °C; ^1^H NMR (400 MHz, CDCl_3_): δ = 7.74 (s, 2H), 7.17 (d, *J* = 8.2 Hz, 2H), 7.00–6.94 (m, 4H), 3.91 (s, 6H), 3.87 (s, 6H), 3.74 (s, 4H), 2.45 (s, 3H) ppm; ^13^C NMR (100 MHz, CDCl_3_): δ = 186.47, 153.54, 151.00, 140.36, 135.56, 134.25, 133.28, 122.42, 122.34, 114.64, 77.00, 56.80, 55.92, 55.56, 45.61 ppm; FTIR (ATR, ṽ): 2950, 1754, 1687, 1601, 1517, 1439, 1315, 1266 cm^−1^; HRMS (ESI): calcd. for: C_26_H_28_NO_9_ [M + H]^+^ 498.1759; found 498.17412.

**(E)-2-Methoxy-4-(3-(1-methyl-4-oxopiperidin-3-yl)-3-oxoprop-1-en-1-yl)phenyl methyl carbonate** (**3**)

**Typical procedure 1** for acylation of piperydone and *α*,*β*-unsaturated piperydones.

To a dry, argon-filled Schlenk tube, anhydrous THF (50 mL) and diisopropyl amine (1.05 mL, 7.41 mmol) were added. The solution was cooled to 0 °C and then *n*-butyllithium was added (2.5 M solution in hexanes, 2.97 mL, 7.41mmol). After 30 min, *N*-methyl-4-piperidone (0.83 mL, 6.74 mmol) was added dropwise to the reaction. The reaction was mixed at 0 °C for 60 min and then cooled to −78 °C, and after an additional 15 min, a solution of (*E*)-4-(3-cyano-3-oxoprop-1-en-1-yl)-2-methoxyphenyl methyl carbonate (1.936 g, 7.41 mmol) in dry THF (10 mL) was added. The reaction was stirred at −78 °C for 30 min and then quenched with a 10% aqueous solution of citric acid (15 mL). The reaction mixture was pureed in water (100 mL) and extracted with dichloromethane (4 × 50 mL). The combined organic extracts were dried with Na_2_SO_4_, filtered, and concentrated under a vacuum. The product was predicated by flash column chromatography (EtOH:DCM:AcOEt, 5:25:70) to yield a white solid (1.192 g, 51%).

Mp: 113–115 °C; ^1^H NMR (400 MHz, CDCl_3_): δ = 16.46 (s, 1H), 7.67 (d, *J* = 15.5 Hz, 1H), 7.25–7.03 (m, 5H), 6.66 (d, *J* = 15.5 Hz, 1H), 3.91 (s, 3H), 3.90 (s, 3H), 3.41 (s, 2H), 2.67 (t, *J* = 5.8 Hz, 2H), 2.59 (t, *J* = 5.8 Hz, 2H), 2.49 (s, 3H) ppm; ^13^C NMR (100 MHz, CDCl_3_): δ = 191.63, 177.57, 153.48, 151.36, 141.36, 140.88, 134.28, 122.74, 120.57, 119.04, 112.14, 105.83, 56.02, 55.59, 53.32, 51.29, 46.02, 34.18 ppm; FTIR (ATR, ṽ): 2945, 2837, 1763, 1629, 1597, 1512, 1439, 1253 cm^−1^; HRMS (ESI): calcd. for: C_18_H_22_NO_6_ [M + H]^+^ 348.1442; found 348.14356.

**3-((E)-benzylidene)-5-cinnamoyl-1-methylpiperidin-4-one** (**4**)

Compound **4** was prepared according to **Typical procedure 1** from (*E*)-3-benzylideno-1-metylopiperyd-4-one (0.392 g, 1.95 mmol) [55] and (*E*)-cinnamoyl cyanide (0.346 g, 2.2 mmol) [25] and purified by flash column chromatography (AcOEt:Hex, 30:70) to yield a pink solid (0.588 g, 91%).

Mp: 115–118 °C; ^1^H NMR (400 MHz, CDCl_3_): δ = 16.79 (s, 1H), 7.80 (d, *J* = 15.5 Hz, 1H), 7.75 (s, 1H), 7.63–7.58 (m, 2H), 7.46–7.33 (m, 8H), 6.90 (d, *J* = 15.5 Hz, 1H), 3.62 (s, 2H), 3.58 (s, 2H), 2.51 (s, 3H) ppm; ^13^C NMR (100 MHz, CDCl_3_): δ = 182.36, 177.33, 142.30, 135.32, 134.85, 133.27, 130.28, 130.20, 129.90, 128.77, 128.46, 128.34, 128.20, 119.27, 106.87, 54.82, 53.07, 45.34 ppm; FTIR (ATR, ṽ): 3063, 2755, 1619, 1575, 1545, 1441,1411, 1288 cm^−1^; HRMS (ESI): calcd. for: C_22_H_22_NO_2_ [M + H]^+^ 332.1645; found 332.1650.

**4-((E)-3-(5-((E)-Benzylidene)-1-methyl-4-oxopiperidin-3-yl)-3-oxoprop-1-en-1-yl)-2-methoxyphenyl methyl carbonate** (**8**)

Compound **8** was prepared according to **Typical procedure 1** from (*E*)-3-benzylidene-1-methylpiperidin-4-one (1.610 g, 8.0 mmol) [51] and (*E*)-4-(3-cyano-3-oxoprop-1-en-1-yl)-2-methoxyphenyl methyl carbonate (1.841 g, 7.0 mmol) [25] and purified by flash column chromatography (AcOEt:Toluene, 50:50) to yield a pink solid (2.470 g, 81%).

Mp: 130–131 °C; ^1^H NMR (400 MHz, CDCl_3_): δ = 16.81 (s, 1H), 7.73 (s, 1H), 7.71 (d, *J* = 15.5 Hz, 1H), 7.41–7.32 (m, 5H), 7.19–7.10 (m, 3H), 6.80 (d, *J* = 15.5 Hz, 1H), 3.91 (s, 3H), 3.90 (s, 3H), 3.59 (s, 2H), 3.54 (s, 2H), 2.49 (s, 3H) ppm; ^13^C NMR (100 MHz, CDCl_3_): δ = 181.80, 177.85, 153.44, 151.33, 141.42, 141.37, 135.37, 134.20, 133.40, 130.47, 129.97, 128.53, 128.39, 122.71, 120.73, 119.77, 112.16, 107.09, 55.97, 55.56, 55.02, 53.21, 45.54 ppm; FTIR (ATR, ṽ): 2935, 1766, 1620, 1587, 1506, 1438, 1416, 1249 cm^−1^; HRMS (ESI): calcd. for: C_25_H_26_NO_6_ [M + H]^+^ 436.1755; found 436.17546.

**4-((E)-(5-Cinnamoyl-1-methyl-4-oxopiperidin-3-ylidene)methyl)-2-methoxyphenyl methyl carbonate** (**6**)

Compound **6** was prepared according to **Typical procedure 1** from **2** (2.451 g, 8.0 mmol) and (*E*)-cinnamoyl cyanide (1.392 g, 8.8 mmol) [25] and purified by flash column chromatography (AcOEt:Toluene, 40:60) to yield a pink solid (1.982 g, 57%).

Mp: 134–135 °C; ^1^H NMR (400 MHz, CDCl_3_): δ = 16.74 (s, 1H), 7.80 (d, *J* = 15.5 Hz, 1H), 7.69 (s, 1H), 7.63–7.58 (m, 2H), 7.42–7.37 (m, 3H), 7.18 (d, *J* = 8.1 Hz, 1H), 6.96–6.87 (m, 3H), 3.94 (s, 3H), 3.89 (s, 3H), 3.60 (s, 2H), 3.58 (s, 2H), 2.51 (s, 3H) ppm; ^13^C NMR (100 MHz, CDCl_3_): δ = 182.74, 176.87, 153.61, 150.92, 142.49, 139.98, 134.88, 134.64, 132.18, 130.96, 130.29, 128.83, 128.26, 122.23, 122.12, 119.30, 114.18, 107.18, 55.90, 55.54, 54.89, 53.23, 45.53 ppm; FTIR (ATR, ṽ): 2935, 1763, 1624, 1598, 1577, 1511, 1446, 1408, 1315, 1288 cm^−1^, HRMS (ESI): calcd. for: C_25_H_26_NO_6_ [M + H]^+^ 436.1755; found 436.17576.

**2-Methoxy-4-((E)-(5-((E)-3-(3-methoxy-4-((methoxycarbonyl)oxy)phenyl)acryloyl)-1-methyl-4-oxopiperidin-3-ylidene)methyl)phenyl methyl carbonate** (**12**)

Compound **12** was prepared according to **Typical procedure 1** from **2** (2.110 g, 6.9 mmol) and (*E*)-4-(3-cyano-3-oxoprop-1-en-1-yl)-2-methoxyphenyl methyl carbonate (1.983 g, 7.59 mmol) [25] and purified by flash column chromatography (MeOH:DCM; 5:95) to yield a pink solid (1.325 g, 36%).

Mp: 104–108 °C; ^1^H NMR (400 MHz, CDCl_3_): δ = 16.74 (s, 1H), 7.71 (d, *J* = 15.5 Hz, 1H), 7.66 (s, 1H), 7.20–7.11 (m, 4H), 6.94–6.89 (m, 2H), 6.79 (d, *J* = 15.5 Hz, 1H), 3.92 (s, 3H), 3.91 (s, 3H), 3.90 (s, 3H), 3.86 (s, 3H), 3.57 (s, 2H), 3.54 (s, 2H), 2.49 (s, 3H) ppm; ^13^C NMR (100 MHz, CDCl_3_): δ = 182.16, 177.34, 153.62, 153.45, 151.36, 150.95, 141.57, 141.48, 140.04, 134.63, 134.17, 132.38, 130.99, 122.74, 122.26, 122.16, 120.75, 119.75, 114.22, 112.21, 107.24, 55.99, 55.93, 55.57, 55.55, 54.95, 53.21, 45.54 ppm; FTIR (ATR, ṽ): 2949, 2839, 1761, 1618, 1507, 1438, 1250, 1204, 1159 cm^−1^; HRMS (ESI): calcd. for: C_28_H_30_NO_10_ [M + H]^+^ 540.1864; found 540.18483.

**3-Cinnamoyl-5-((E)-4-hydroxy-3-methoxybenzylidene)-1-methylpiperidin-4-one** (**5**)

**Typical procedure 2** for the hydrolysis of carbamate protective group.

To compound **6** (0.085 g, 0.195 mmol), methanol (2 mL) and DCM were added in amounts for complete dissolution (3 mL). The solution was cooled to 0 °C, then a solution of ammonia in water (10%, 1 mL) was added after 20 min. The reaction was heated to room temperature and left overnight (monitored by TLC). The next day, 20 mL of water and a 10% water solution of citric acid to pH = 5 were added. The obtained mixture was extracted with DCM (3 × 20 mL), and the combined organic extracts were dried over filtered Na_2_SO_4_ and concentrated under vacuum. The product was predicated by flash column chromatography (EtOH:DCM:AcOEt; 2:28:70) to yield an orange solid (0.046 g, 62%).

Mp: 145–147 °C; ^1^H NMR (400 MHz, CDCl_3_): δ = 16.92 (s, 1H), 7.77 (d, *J* = 15.5 Hz, 1H), 7.67 (s, 1H), 7.61–7.57 (m, 2H), 7.42–7.37 (m, 3H), 6.95–6.85 (m, 4H), 6.37 (s, 1H), 3.88 (s, 3H), 3.64 (s, 2H), 3.58 (s, 2H), 2.52 (s, 3H) ppm; ^13^C NMR (100 MHz, CDCl_3_): δ = 181.20, 178.74, 146.66, 146.53, 141.99, 135.09, 133.91, 130.20, 128.88, 128.51, 128.24, 127.83, 123.86, 119.34, 114.66, 113.05, 106.62, 55.87, 55.28, 53.20, 45.50. ppm; FTIR (ATR, ṽ): 3001, 2949, 2910, 1617, 1575, 1509, 1446, 1422, 1289, 1277, 1132, 1120 cm^−1^; HRMS (ESI): calcd. for: C_23_H_24_NO_4_ [M + H]^+^ 378.1700; found 378.17102.

**(E)-3-Benzylidene-5-((E)-3-(4-hydroxy-3-methoxyphenyl)acryloyl)-1-methylpiperidin-4-one** (**7**)

Compound **7** was prepared according to **Typical procedure 2** from **8** (1.151 g, 2.64 mmol) and purified by flash column chromatography (EtOH:DCM:AcOEt; 2:28:70) to yield a pink solid (0.477 g, 48%).

Mp: 172–174 °C; ^1^H NMR (400 MHz, CDCl_3_): δ = 16.88 (s, 1H), 7.78–7.69 (m, 2H), 7.45–7.39 (m, 2H), 7.38–7.30 (m, 3H), 7.19 (dd, *J* = 8.3, 1.9 Hz, 1H), 7.05 (d, *J* = 1.9 Hz, 1H), 6.94 (d, *J* = 8.2 Hz, 1H), 6.72 (d, *J* = 15.4 Hz, 1H), 6.15 (br s, 1H) 3.96 (s, 3H), 3.62 (s, 2H), 3.58 (s, 2H), 2.51 (s, 3H) ppm; ^13^C NMR (100 MHz, CDCl_3_): δ = 183.50, 176.27, 148.41, 146.95, 142.99, 135.55, 133.00, 130.34, 129.94, 128.44, 128.41, 127.45, 122.92, 116.74, 115.11, 110.39, 106.59, 55.93, 54.89, 53.25, 45.43 ppm; FTIR (ATR, ṽ): 2916, 1617, 1590, 1577, 1506, 1426, 1270, 1158, 1121 cm^−1^; HRMS (ESI): calcd. for: C_23_H_24_NO_4_ [M + H]^+^ 378.1700; found 378.17111.

**(3E,5E)-5-(4-Hydroksy-3-metoksybenzylideno)-3-[3-(4-hydroksy-3-metoksy)prop-2-enylo]-1-metylopiperyd-4-on** (**11**)

Compound **11** was prepared according to **Typical procedure 2** from **12** (0.065 g, 0.12 mmol) and purified by flash column chromatography (EtOH/DCM/AcOEt, 5:25:70) followed by recrystallization from DCM to yield a pink solid (0.018 g, 35%).

Mp: 174–176 °C; ^1^H NMR (400 MHz, CDCl_3_): δ = 16.98 (s, 1H), 7.72 (d, *J* = 15.4 Hz, 1H), 7.66 (s, 1H), 7.21–7.18 (m, 1H), 7.05 (s, 1H), 6.99–6.85 (m, 4H), 6.71 (d, *J* = 15.4 Hz, 1H), 5.98 (br s, 2H), 3.97 (s, 3H), 3.92 (s, 3H), 3.64 (s, 2H), 3.58 (s, 2H), 2.52 (s, 3H) ppm; ^13^C NMR (100 MHz, CDCl_3_ + CD_3_OD): δ = 182.49, 176.35, 148.87, 147.49, 147.10, 147.09, 143.08, 133.92, 127.40, 127.29, 127.09, 123.72, 123.02, 116.24, 115.26, 114.96, 113.50, 110.74, 105.72, 77.06, 55.76, 55.68, 54.97, 52.94, 44.98 ppm; FTIR (ATR, ṽ): 2959, 1767, 1693, 1570, 1513, 1446, 1412, 1278 cm^−1^; HRMS (ESI): calcd. for: C_24_H_26_NO_6_ [M + H]^+^ 424.1755; found 424.17516.

**(3E,5E)-3,5-Bis(4-hydroksy-3-metoksyfenylo)-1-metylopiperyd-4-on** (**9**)

To *N*-metylopiperyd-4-one (1.357 g, 12.02 mmol), vanillin (4.226 g, 27.78 mmol) and acetic acid (99%, 4 mL) were added. The reaction was heated to 45 °C for two days then the temperature was increased to 100 °C and heated for an additional day. After cooling to room temperature, 2 mL of concentrated hydrochloric acid was added, and the solution was evaporated to dryness under vacuum. To the obtained residue, water and NaHCO_3_ at a neutral pH were added and extracted with DCM (4 × 50 mL). Combined organic extracts were dried over Na_2_SO_4_, filtered, and concentrated under a vacuum. The product was predicated by flash column chromatography (MeOH:DCM; 5:95) to yield a brown solid (1.27 g, 22%).

Mp: 217–218 °C (lit. 195–197; Youssef, 2004); ^1^H NMR (400 MHz, DMSO): δ = 9.61 (s, H), 7.53 (s, 2H), 7.07 (s, 2H), 6.99–6.85 (m, 4H), 3.82 (s, 6H), 3.71 (br s, 4H), 2.40 (s, 3H) ppm. NMR data were in agreement with those reported in literature [26].

### 4.3. The Nitro Blue Tetrazolium (NBT) Test

Superoxide anions have been generated in the solution by dissolving 6.5 mg of KO_2_ powder and 90 mg 18-crown-6-ether in DMSO (50 mL). The solution of NBT has been prepared by dissolving 10 mg of NBT in 10 mL of DMSO. The control sample contained 1.5 mL of superoxide anions solution (DMSO), 0.5 mL of DMSO, and 0.1 mL of nitro blue tetrazolium solution. The solutions of curcumin and its analogues have been prepared in DMSO. The cuvette with the tested sample contained 1.5 mL of the superoxide anions solution, 0.5 mL of the antioxidant solution (curcumin and its analogues), and 0.1 mL of the nitro blue tetrazolium solution. The changes in absorbance were monitored spectrophotometrically at 560 nm. The starting concentrations of the antioxidant solutions in the measuring cuvettes were selected so that possible products formed by curcumin as well as its analogues and superoxide anion radicals did not interfere with the absorbance at 560 nm. Each measurement was repeated 3 times. The value of the superoxide anion scavenging by the tested compounds was calculated using the appropriate formula:

Scavenging = (A_0_ − A/A_0_) × 100% (1)

where A_0_ denotes the absorbance of the control sample and A is the absorbance of the tested sample.

### 4.4. Cell Lines and Culture

The breast cancer MCF-7 and MDA-MB-231 cell lines and human keratinocyte HaCaT cell line were obtained from American Type Culture Collection (ATCC, Manassas, VA, USA). The cell lines were cultured in Dulbecco’s Modified Eagle’s Medium supplemented with 10% fetal bovine serum (FBS) and 1% penicillin/streptomycin. The cell culture was maintained at 37 °C in an atmosphere containing 5% CO_2_.

### 4.5. Cell Viability Assay

A cell viability assay (MTT assay) was performed according to the general protocol. Briefly, the cells (1.2 × 10^6^ cells/mL) were treated with solvent (referred to as control) or treated with solutions of curcumin or its derivatives at concentrations ranging 7.81–100 µM. The solutions of compounds were prepared in a culture medium with 1% penicillin/streptomycin and without fatal bovine serum. After 24 h, the solution of 0.5 mg/mL MTT (3-[4,5-dimethylthiazol-2-yl]-2,5-diphenyl-tetrazolium bromide) was added. When the purple precipitate was clearly visible under the microscope, 100 µL of DMSO was added to each well. The absorbance at 540 nm was determined using a microplate reader (Biogenet, Jozefow, Poland) and DigiRead Communication Software (Asys Hitech GmbH, Eugendorf, Austria). The data were expressed as the percentage of untreated cells (control).

### 4.6. Flow Cytometry

Flow cytometry was used to analyze the generation of intracellular ROS. Briefly, the MCF-7, MDA-MB-231, and HaCat cell lines were seeded into 6-well plates at a density of 10^6^ cells per plate. Overnight, cells were untreated (as control) or treated with curcumin, compound **5,** and compound **6** in concentrations of 6.25 and 25 µM for 24 h. After the incubation time, cells were exposed to 2′,7′-dichlorofluorescein for 30 min, and the next cells were collected. A flow cytometry test measured the oxidized form of 2′,7′-dichlorofluorescein (excitation wavelength: 480 nm; emission wavelength: 525 nm). The data were expressed as the percentage of untreated cells.

### 4.7. Western Blot

The level of PTP1B phosphatase was determined by the Western blot technique. Briefly, the MCF-7 and MDA-MB-231 cell lines were non-treated (control) or treated with curcumin, compound **5**, and compound **6** in concentrations of 25 µM, for 24 h, then the cells were harvested, centrifuged, and lysed. The protein concentration was determined using the Bradford reagent [56]. The proteins were separated on a 4–20% gradient of polyacrylamide gel by electrophoresis (Bio-Rad Laboratories, Hercules, CA, USA). The separated proteins were transferred to a methanol-activated PVDF membrane in the TBE buffer using a semi-dry transfer device (GE Healthcare, Chicago, IL, USA). Membranes were incubated with primary PTP1B antibodies (Cell Signaling, Danvers, MA, USA), and overnight, with horseradish peroxidase (HRP)-conjugated secondary antibodies. Visualization was performed using chemiluminescence enhanced with a chemiluminescence reagent according to the manufacturer’s protocol. The signal was read using ImageQuant LAS 500 (GE Healthcare, Chicago, IL, USA). Protein levels were quantified using densitometry analysis by the ImageJ program [57]. The results were normalized to β-actin.

### 4.8. Real-Time Microscopy

To obtain real-time effects for the tested compounds against the MDA-MB-231 cell line, the CytoSMART^®^ Lux3 FL (CytoSMART technologies B.V, Lonza, Basel, Switzerland) was used. MDA-MB-231 cells were seeded into a ⌀10 cm plate. When the cell confluence was 60%, cells were rinsed with phosphate-buffered saline, and 5 mL of the medium with 10 µL of 0.01 mg/mL propidium iodide (PI) (to visualization late apoptotic/necrotic cells) and curcumin or compound **5** at the concentration of 25 µM was added. The prepared sample was immediately placed on the CytoSMART^®^ Lux3 FL in the CO_2_ incubator and the image from the microscope was observed. The experiment was carried out used brightfield, green fluorescence (excitation: 452/45 nm; emission: 512/23 nm), and red fluorescence (excitation: 561/14 nm; emission: 630/90 nm) in parallel, or brightfield and red fluorescence in parallel. The snapshot was automatically performed with an interval of 5 min for 24 h in the CO_2_ incubator and collected on the cloud.cytosmart.com.

### 4.9. Recombinant PTP1B Assay

The analysis was performed in 96-well plates. Briefly, the final concentration of PTP1B phosphatase in reaction samples was 0.8 μg/mL (10 nM). The enzyme was treated with solvent (referred as control) or treated with the solution of curcumin and its derivatives in a concentration of 100 µM. The enzymatic activities of PTP1B phosphatase were measured using 1 mM chromogenic substrate *para*-nitrophenyl phosphate (*p*NPP) in 10 mM HEPES buffer pH 7.4, at 37 °C. The increase in absorbance (due to *para*-nitrophenol formation) is linearly proportional to the enzymatic activity concentration (with excessive substrate, i.e., zero-order kinetics) and was assessed at 405 nm on the microplate reader Jupiter (Biogenet, Jozefow, Poland) using DigiRead Communication Software (Asys Hitech GmbH, Eugendorf, Austria). The data were expressed as the percentage of the untreated enzyme (control).

### 4.10. Molecular Docking

Protein tyrosine phosphatase 1B (PTP1B) crystal structure 1T48 (rcsb.org/structure/1T48) was selected for docking simulations. Molecular modelling studies were performed using Small-Molecule Drug Discovery Suite (Schrödinger LLC, New York, NY). Compound 6, BB3, and curcumin structures were optimized using the LigPrep tool, generating the most possible protonation states in the pH range of 7 ± 0.2 [58]. The proper protonation states were additionally verified using MarvinSketch software (Marvin version 19.4.0, ChemAxon, chemaxon.com). Crystal structure 1T48 was refined using Protein Preparation Wizard [59]: Hydrogen atoms were added, and the energy of the whole system was minimized using an OPLS3e force field [60]. Induced-fit docking (IFD) was used to dock compound **6** and determine its putative binding mode within PTP1B [61]. The grid box centroid was set automatically after selecting all the amino acids located up to 6 Å from BB3 in its initial, crystallized position. No constraints were applied during IFD. As an output, the PTP1B-compound **6** complex was obtained and analyzed, based on the observed molecular interactions. Then, redocking of BB3 was performed (Glide software, Standard Precision mode) to validate the obtained model [62,63]. RMSD between originally co-crystalized and redocked ligands was calculated. Additional docking of curcumin, a known ligand of PTP1B, was done to compare its binding mode to the one obtained for compound **6**.

## 5. Conclusions

In this research, we present the results of ten new curcumin derivatives featuring a 4-piperidone structure. Nine of these compounds have a 4-piperidone ring incorporated in a 1,3-diketone moiety. Compounds **1**–**3** contain fragments of the curcumin skeleton. Comparing the compounds containing the full curcumin skeleton or only selected elements of it allowed us to determine the fragments of the structure that are crucial for biological activity.

We observed that two compounds, **5** and **6**, exhibited higher cytotoxic potential against breast cancer cell lines than curcumin. The main mechanism of action for these compounds could be the generation of intracellular ROS levels. Moreover, we have observed that a higher level of ROS resulted in the lower level of PTP1B in MCF-7 and MDA-MB-231 cell lines. It is worth noting that compound **6** showed the highest cytotoxic effect, also in low concentrations. Based on computational analysis, we suggest that this effect may be caused by allosteric inhibition of PTP1B.

According to the obtained results, it is important to look for new inhibitors that, by regulating protein tyrosine phosphatases, could have positive effects in preventing as well as treating cancer or autoimmune diseases. The search for novel compounds with a cytotoxic effect against breast cancer cells correlated with the inhibition of PTP1B protein tyrosine phosphatase may contribute to innovative targeted treatment.

## Figures and Tables

**Scheme 1:**
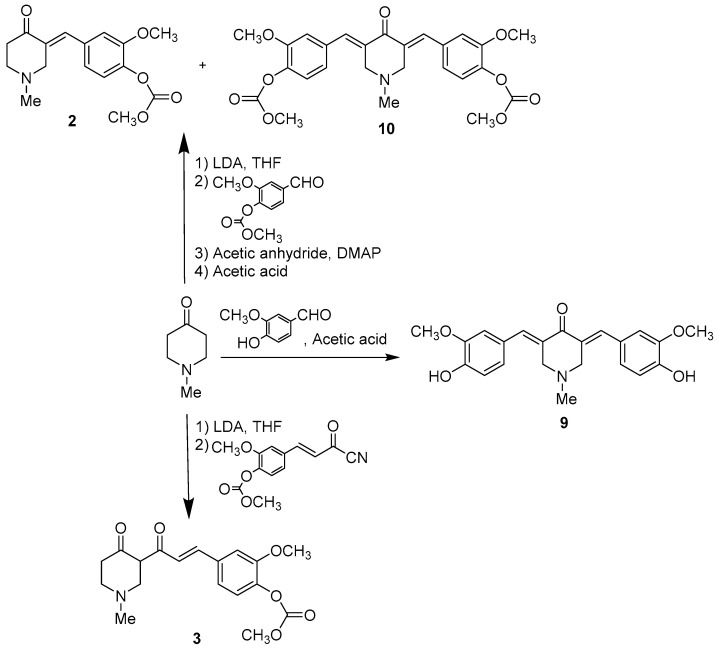
Synthesis of monocarbonyl curcumin analogues and compound **3**.

**Table 1:** The values of percent superoxide anion scavenging by curcumin and its derivatives.

Antioxidant	Concentration [µM]	Scavenging [%]
**Curcumin**	**0.05**	**7**
Compound **1**	1000	5
Compound **2**	1000	11
Compound **3**	0.50	1
Compound **4**	0.05	3
Compound **5**	5.00	5
Compound **6**	0.05	6
Compound **7**	6.25	3
**Compound 8**	**0.05**	**18**
**Compound 9**	**0.08**	**14**
Compound **10**	0.50	3
Compound **11**	0.50	*
**Compound 12**	**0.05**	**9**
l-ascorbic acid [28]	0.05	9.45

* For compound **11**, it was not possible to select the concentration in which the formation of the product with the radical anion would not interfere with the measurement of absorbance at 560 nm.

**Table 2:** IC_50_ values against MCF-7, MDA-MB-231, and HaCaT cell lines for curcumin and its new derivatives.

	IC_5O_ [µM] ± SD			Selectivity Index (SI)	
	MCF-7	MDA-MB-231	HaCaT	HaCat/MCF-7	HaCaT/MDA-MB-231
**Curcumin**	**37.36 ± 1.88**	**57.07 ± 6.23**	**19.44 ± 2.60**	0.52	0.34
Compound **1**	79.38 ± 1.92	>100	60.18 ± 5.82	0.75	<0.60
Compound **2**	>100	60.76 ± 5.04	54.15 ± 3.67	<0.54	0.89
Compound **3**	30.99 ± 6.16	84.23 ± 7.70	41.02 ± 1.43	1.32	0.49
Compound **4**	64.38 ± 11.79	>100	15.31 ± 1.52	0.23	<0.15
**Compound 5**	**25.30 ± 2.54**	**31.86 ± 1.07**	**41.24 ± 3.22**	1.63	1.29
**Compound 6**	**30.51 ± 4.11**	**11.80 ± 1.43**	**30.84 ± 7.63**	1.01	2.61
Compound **7**	42.76 ± 2.18	49.13 ± 6.62	16.78 ± 1.51	0.39	0.34
Compound **8**	39.59 ± 2.18	37.80 ± 2.52	21.94 ± 3.61	0.55	0.58
Compound **9**	>100	>100	10.50 ± 1.44	<0.11	<0.11
Compound **10**	28.27 ± 1.56	>100	8.38 ± 3.80	0.30	<0.08
Compound **11**	32.44 ± 3.44	92.34 ± 2.98	52.79 ± 6.86	1.63	0.57
Compound **12**	>100	71.96 ± 1.82	18.13 ± 3.46	<0.18	0.25
Doxorubicin	0.41 ± 0.75 ^a^	0.60 ± 0.36 ^a^	2.72 ^b^	6.63 ^d^	4.53 ^d^
Tamoxifen	24.76 ± 0.80 ^a^	25.84 ± 1.60 ^a^	22.00 ± 1.02 ^c^	0.89 ^d^	0.85 ^d^

^a^ [29]; ^b^ [30]; ^c^ [31]; ^d^ SI calculated based on literature data.

## Data Availability

Data is contained within the article or supplementary material.

## Supplementary Materials

The following are available online at https://www.mdpi.com/article/10.3390/ijms221910368/s1.
