# Supplementary material for: Curcumin and Its New Derivatives: Correlation between Cytotoxicity against Breast Cancer Cell Lines, Degradation of PTP1B Phosphatase and ROS Generation
Source: Int J Mol Sci. 2021 Sep 26;22(19):10368. doi: 10.3390/ijms221910368 (PMC8508995; doi:10.3390/ijms221910368)

Supplementary Material

Curcumin and its new derivatives: correlation between cytotoxicity against breast cancer cell lines, degradation of PTP1B phosphatase and ROS generation

Tomasz Kostrzewa[1]*, Karol Wołosewicz[2], Marek Jamrozik[3], Joanna Drzeżdżon[4], Julia Siemińska[2,5], Dagmara Jacewicz[4], Magdalena Górska-Ponikowska[1,5,6], Marcin Kołaczkowski[3], Ryszard Łaźny[2], Alicja Kuban-Jankowska[1]*

1. Department of Medical Chemistry, Faculty of Medicine, Medical University of Gdansk, 80-211 Gdansk, Poland; magdalena.gorska-ponikowska@gumed.edu.pl (M.G.-P.)
2. Department of Chemistry, University of Bialystok, Ciolkowskiego 1K, 15-245 Bialystok, Poland; k.wolosewicz@uwb.edu.pl (K.W.); julia.sieminska@umb.edu.pl (J.S.); lazny@uwb.edu.pl (R.Ł.)
3. Department of Medicinal Chemistry, Faculty of Pharmacy, Jagiellonian University Medical College, 30-688 Krakow, Poland
   marek.jamrozik@doctoral.uj.edu.pl (M.J.); marcin.kolaczkowski@uj.edu.pl (M.K.)
4. Department of Environmental Technology, Faculty of Chemistry, University of Gdansk, Wita Stwosza 63, 80-308 Gdansk, Poland;
   joanna.drzezdzon@ug.edu.pl (J.D.); dagmara.jacewicz@ug.edu.pl (D.J.)
5. Metabolomics Laboratory, Clinical Research Center, Medical University of Bialystok, 15-276 Balystok, Poland
6. The Euro-Mediterranean Institute of Science and Technology, 90139 Palermo, Italy;
7. Institute of Biomaterials and Biomolecular Systems, Department of Biophysics, University of Stuttgart,70174 Stuttgart, Germany;
   * Correspondence: tomasz.kostrzewa@gumed.edu.pl (T.K.);
   alicja.kuban-jankowska@gumed.edu.pl (A.K.-J.)

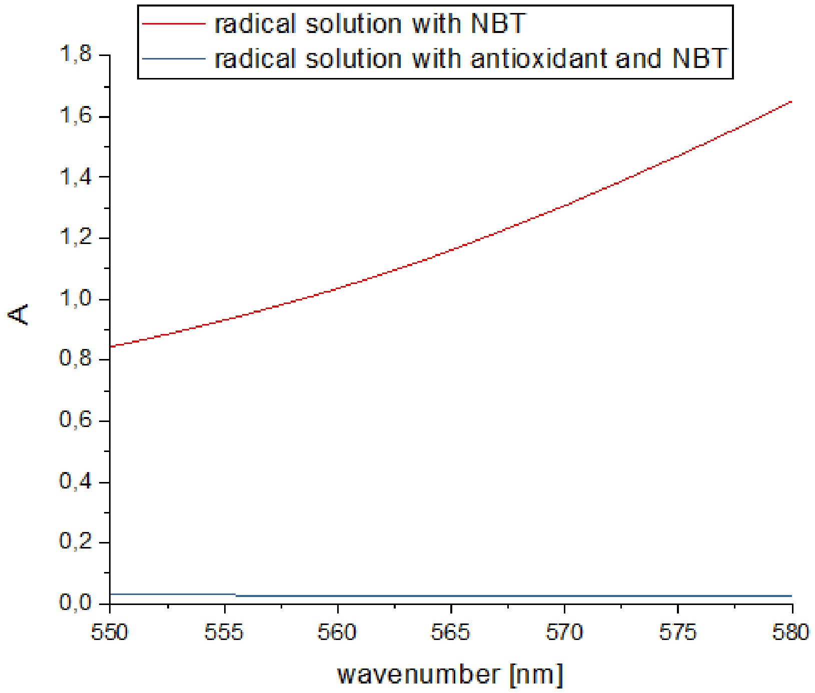

The selected UV-Vis spectra in the range 550-580 nm registered during NBT test for compound 8.

(3*E*)-3-[(4-Hydroxy-3-methoxyphenyl)methylidene]-1-methylpiperidin-4-one (**1**) ^{1}H NMR (400 MHz, $CDCl_3$)

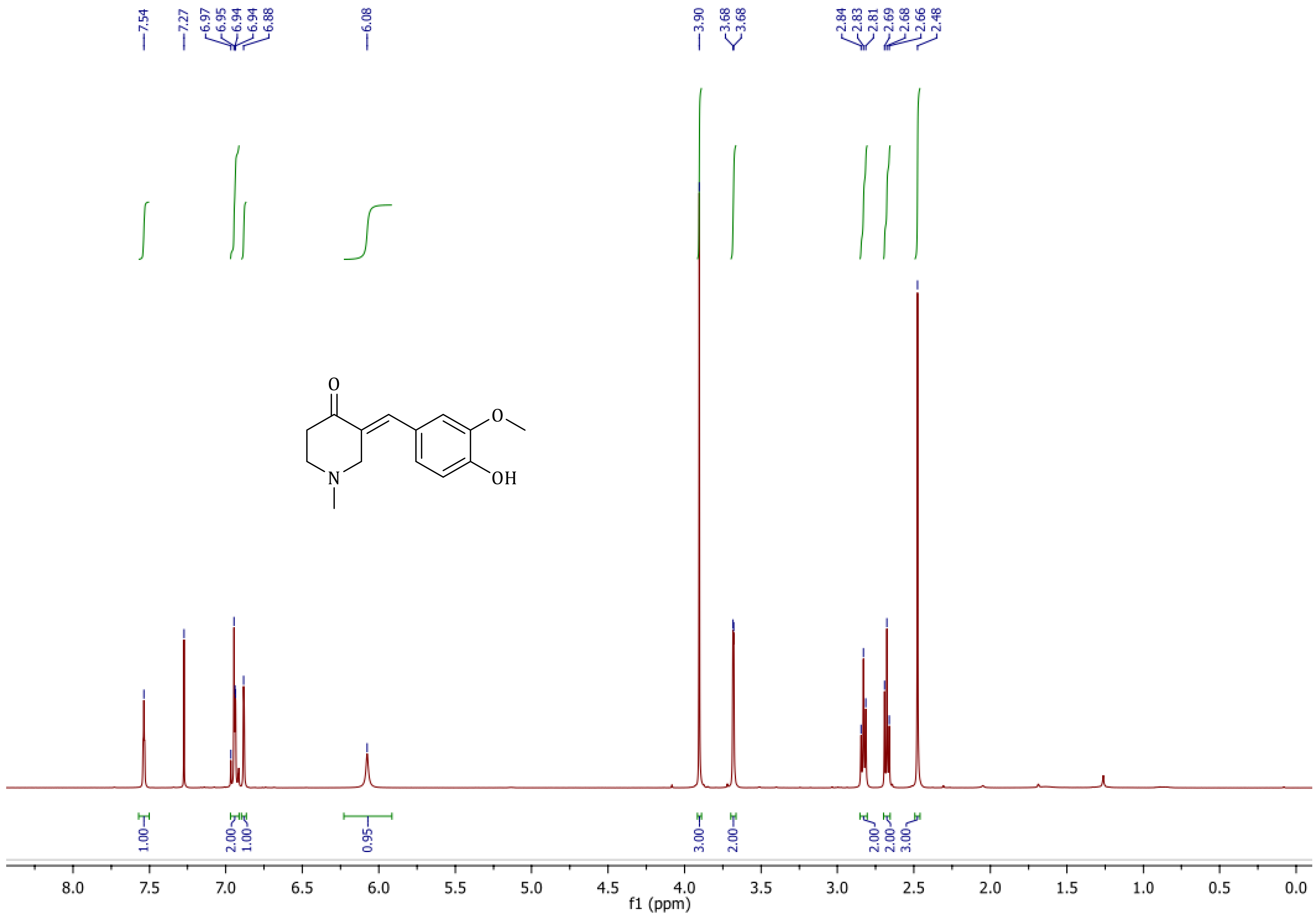

(3*E*)-3-[(4-Hydroxy-3-methoxyphenyl)methylidene]-1-methylpiperidin-4-one (**1**) ^{13}C NMR (100 MHz, $CDCl_3$)

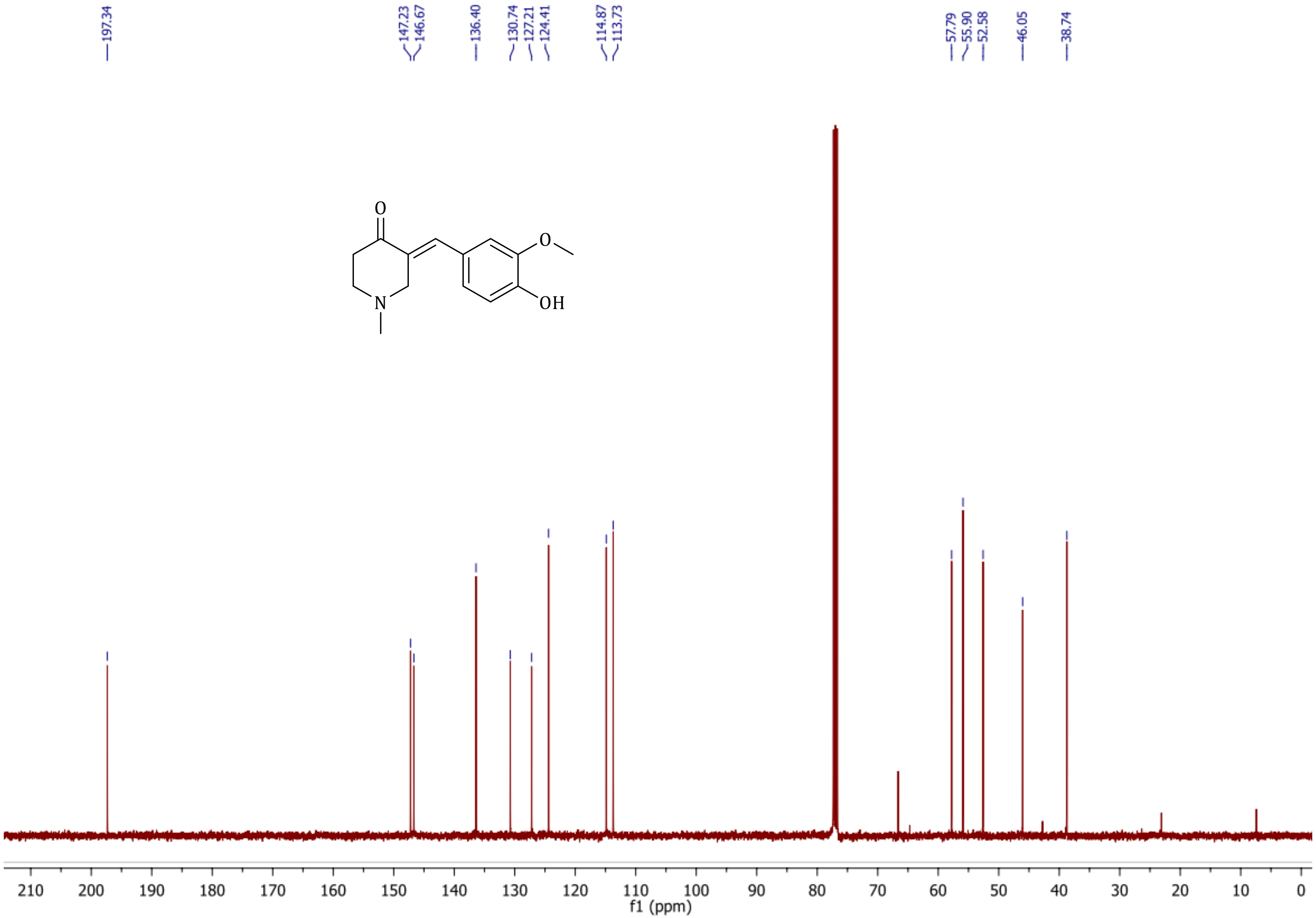

(*E*)-2-Methoxy-4-((1-methyl-4-oxopiperidin-3-ylidene)methyl)phenyl methyl carbonate (**2**) ^{1}H NMR (400 MHz, $CDCl_3$)

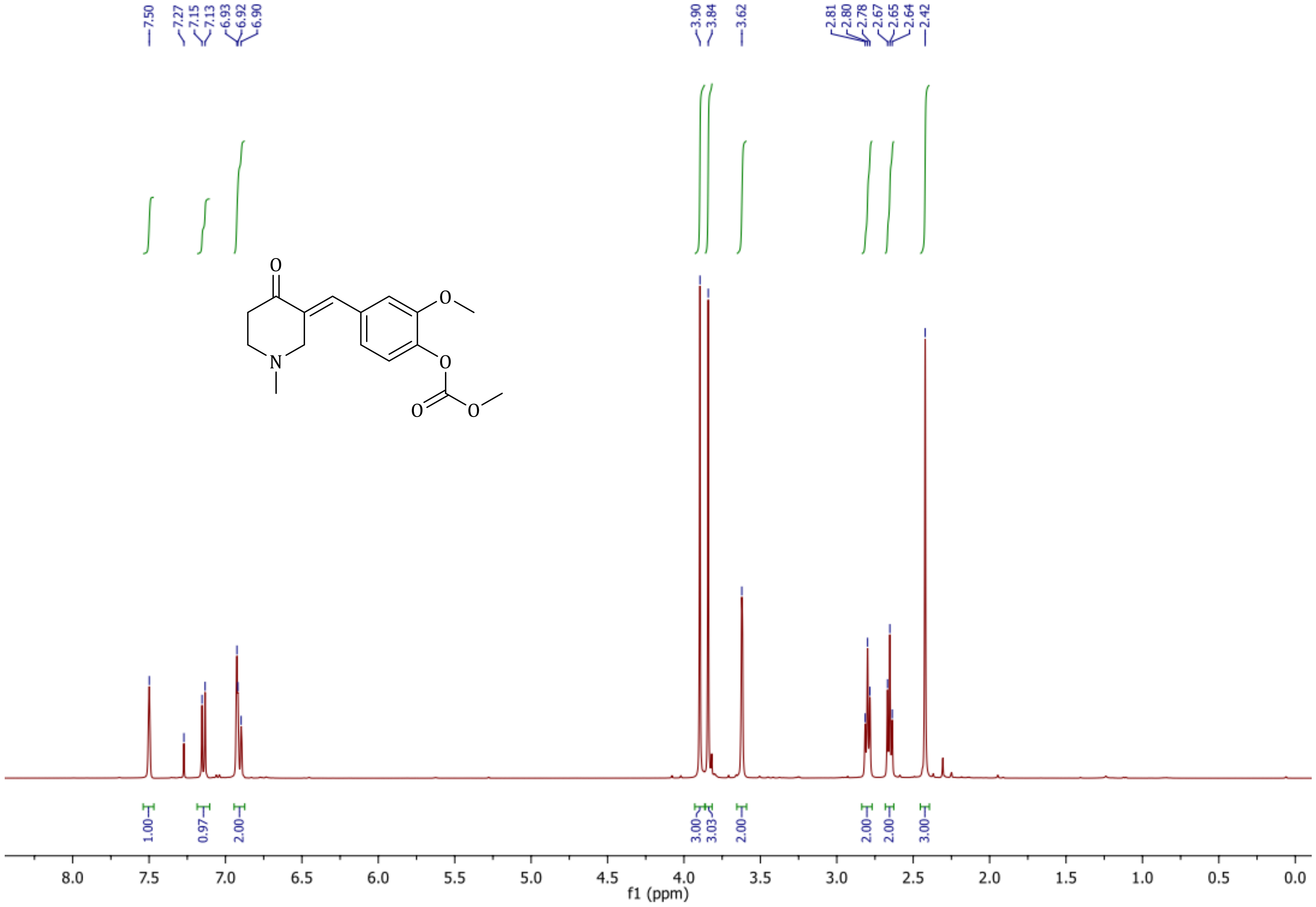

(*E*)-2-Methoxy-4-((1-methyl-4-oxopiperidin-3-ylidene)methyl)phenyl methyl carbonate (**2**) ^{13}C NMR (100 MHz, $CDCl_3$)

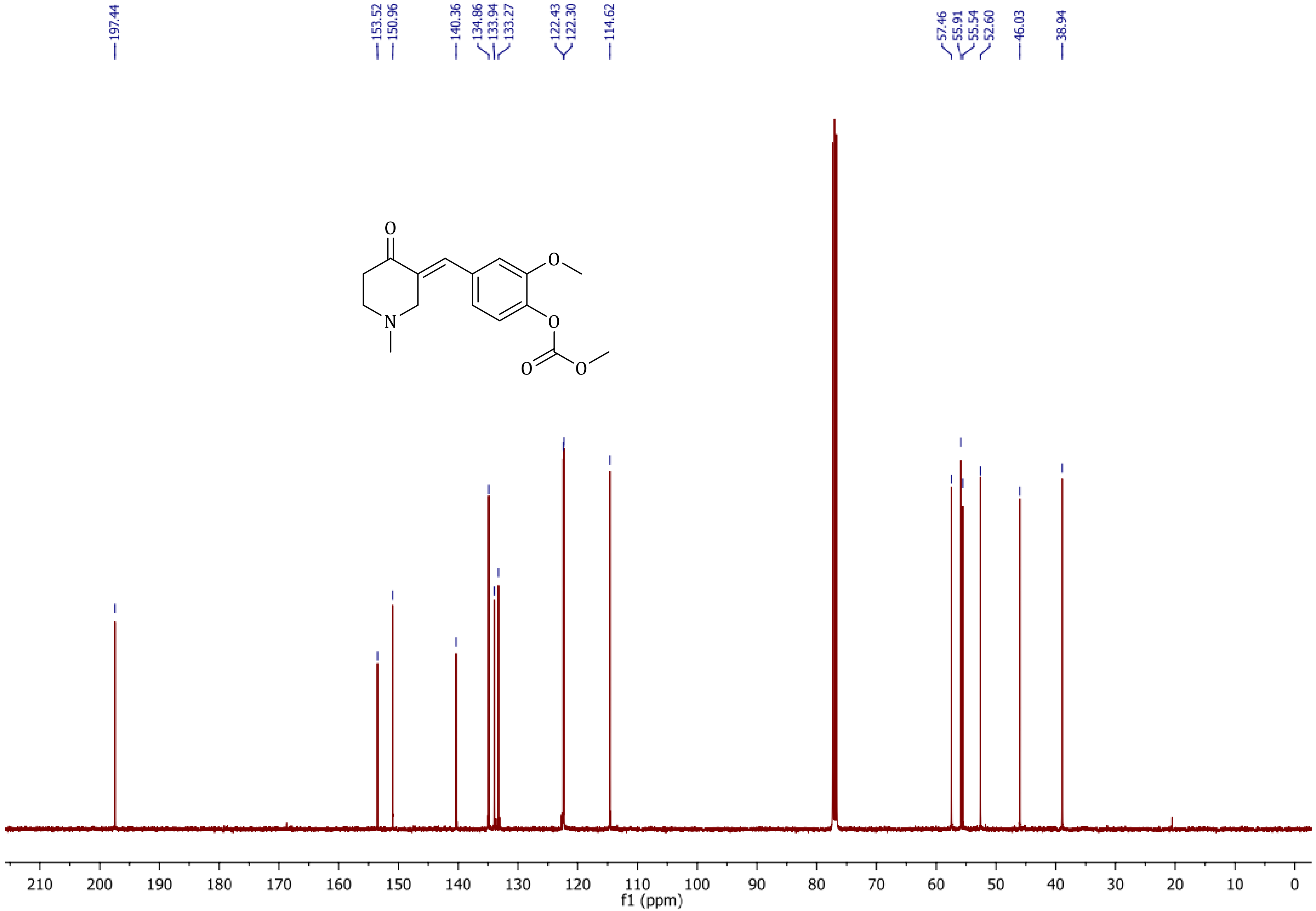

IR spectra of compound **2**; Attenuated Total Reflectance (ATR)

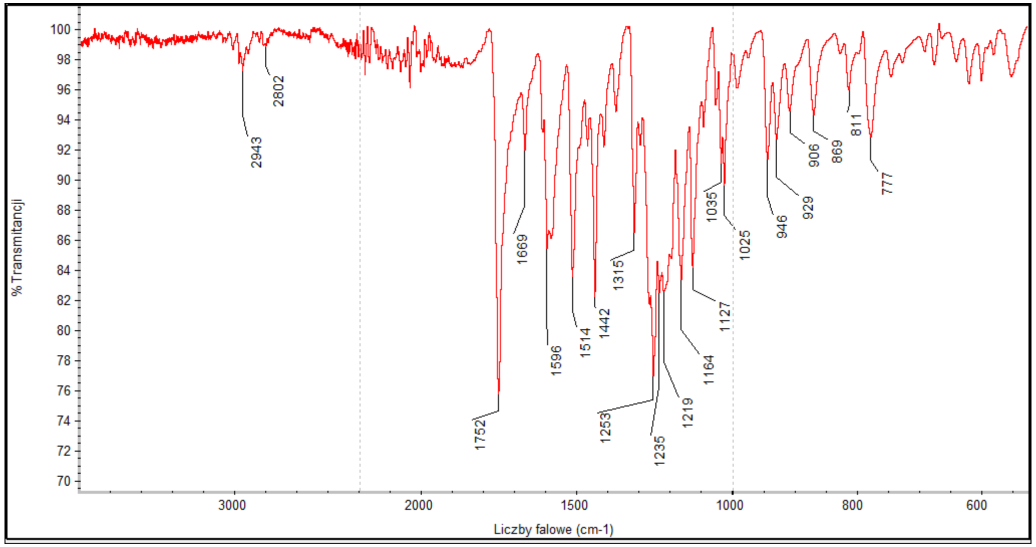

Compound **2** - HRMS (ESI): calcd. for: $C_{16}H_{20}NO_5$ $[M+H]^+$ 306.1336

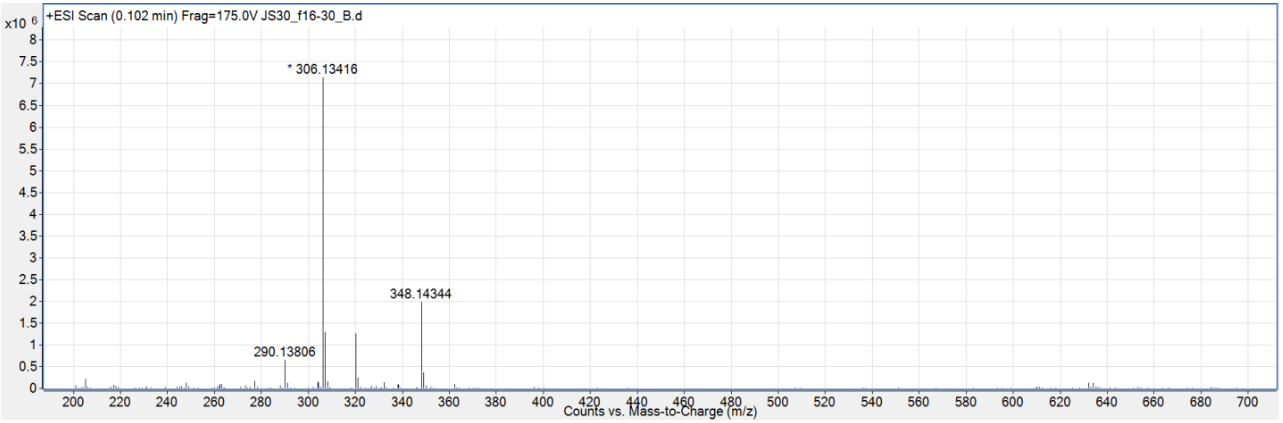

(*E*)-2-Methoxy-4-(3-(1-methyl-4-oxopiperidin-3-yl)-3-oxoprop-1-en-1-yl)phenyl methyl carbonate (**3**) ^{1}H NMR (400 MHz, $CDCl_3$);

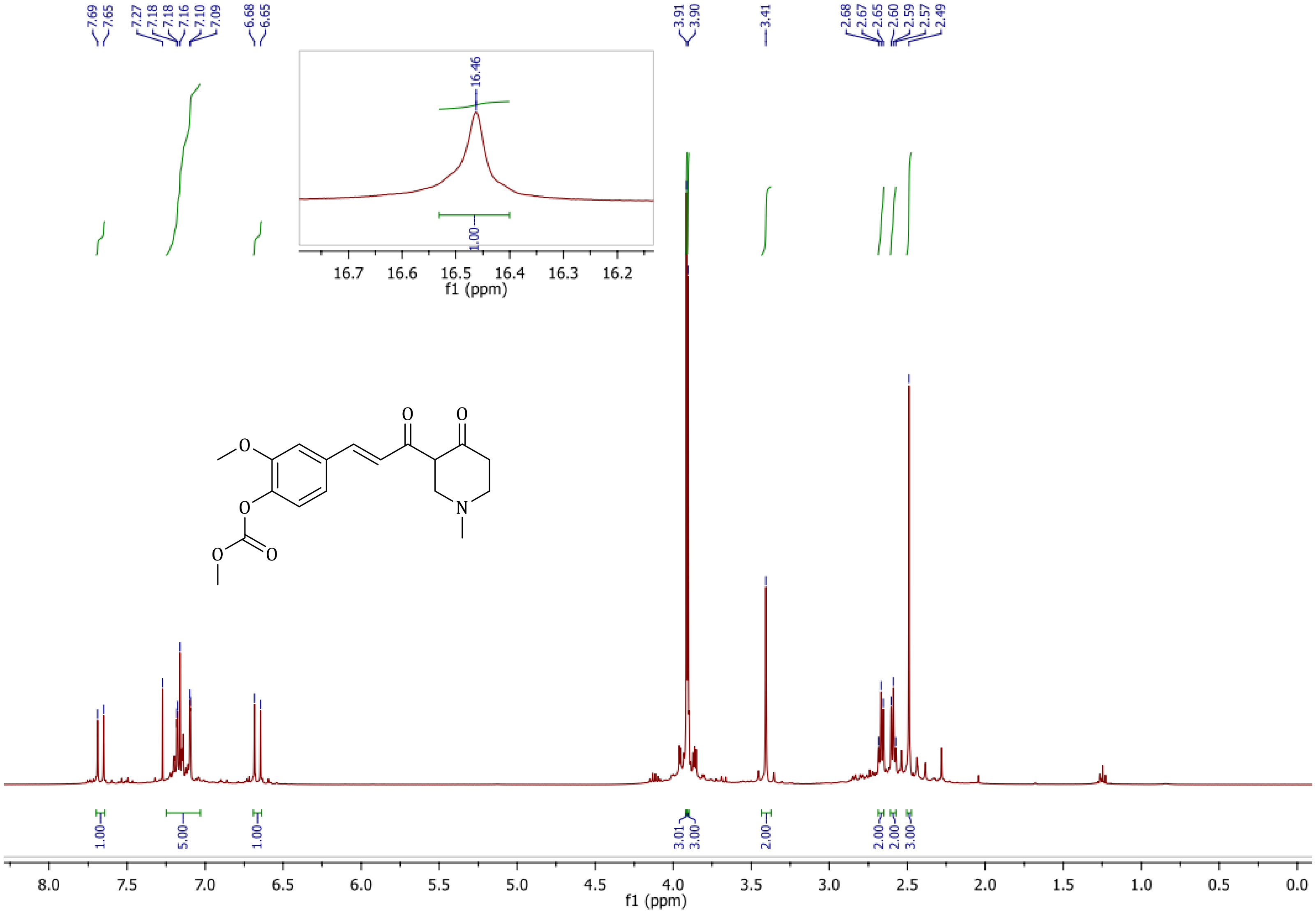

(*E*)-2-Methoxy-4-(3-(1-methyl-4-oxopiperidin-3-yl)-3-oxoprop-1-en-1-yl)phenyl methyl carbonate (**3**) ^{13}C NMR (100 MHz, $CDCl_3$)

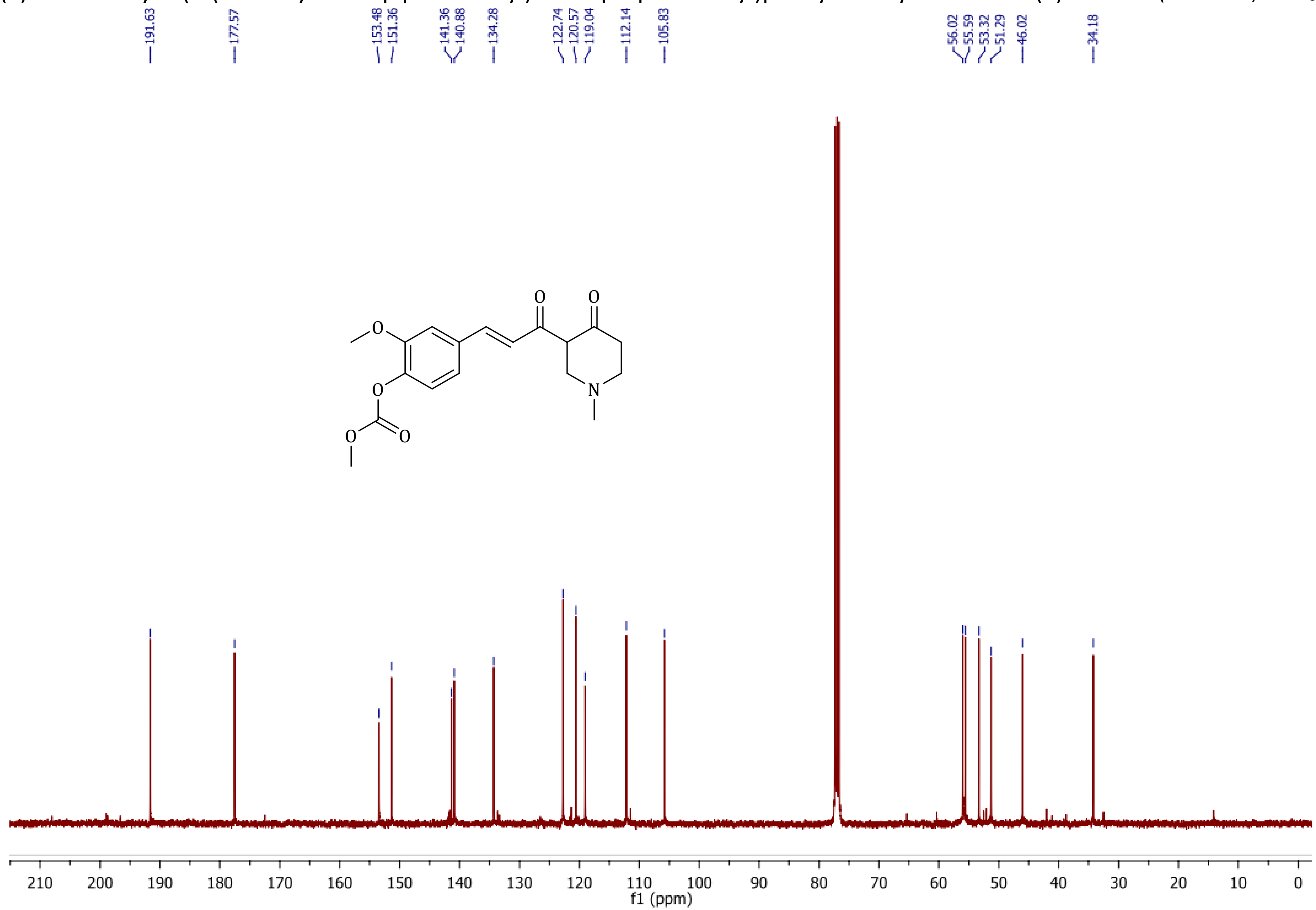

IR spectra of compound **3**; Attenuated Total Reflectance (ATR)

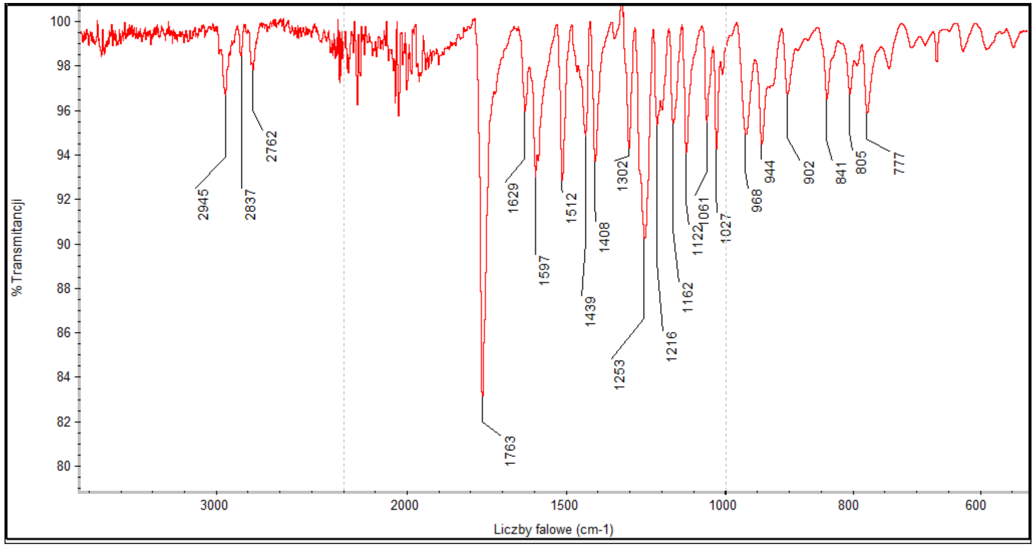

Compound **3** - HRMS (ESI): calcd. for: $C_{18}H_{22}NO_6$ $[M+H]^+$ 348.1442

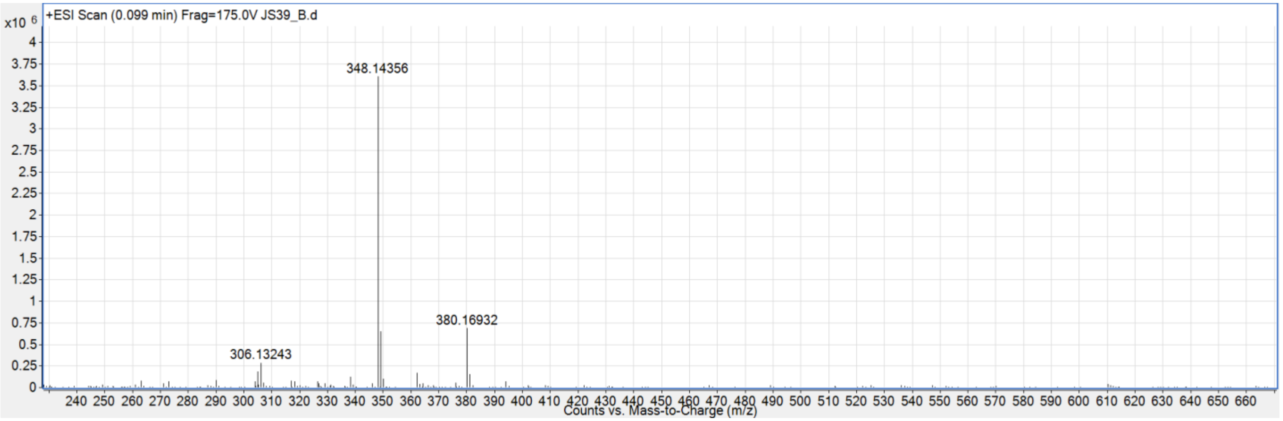

3-((*E*)-benzylidene)-5-cinnamoyl-1-methylpiperidin-4-one (**4**) ^{1}H NMR (400 MHz, $CDCl_3$)

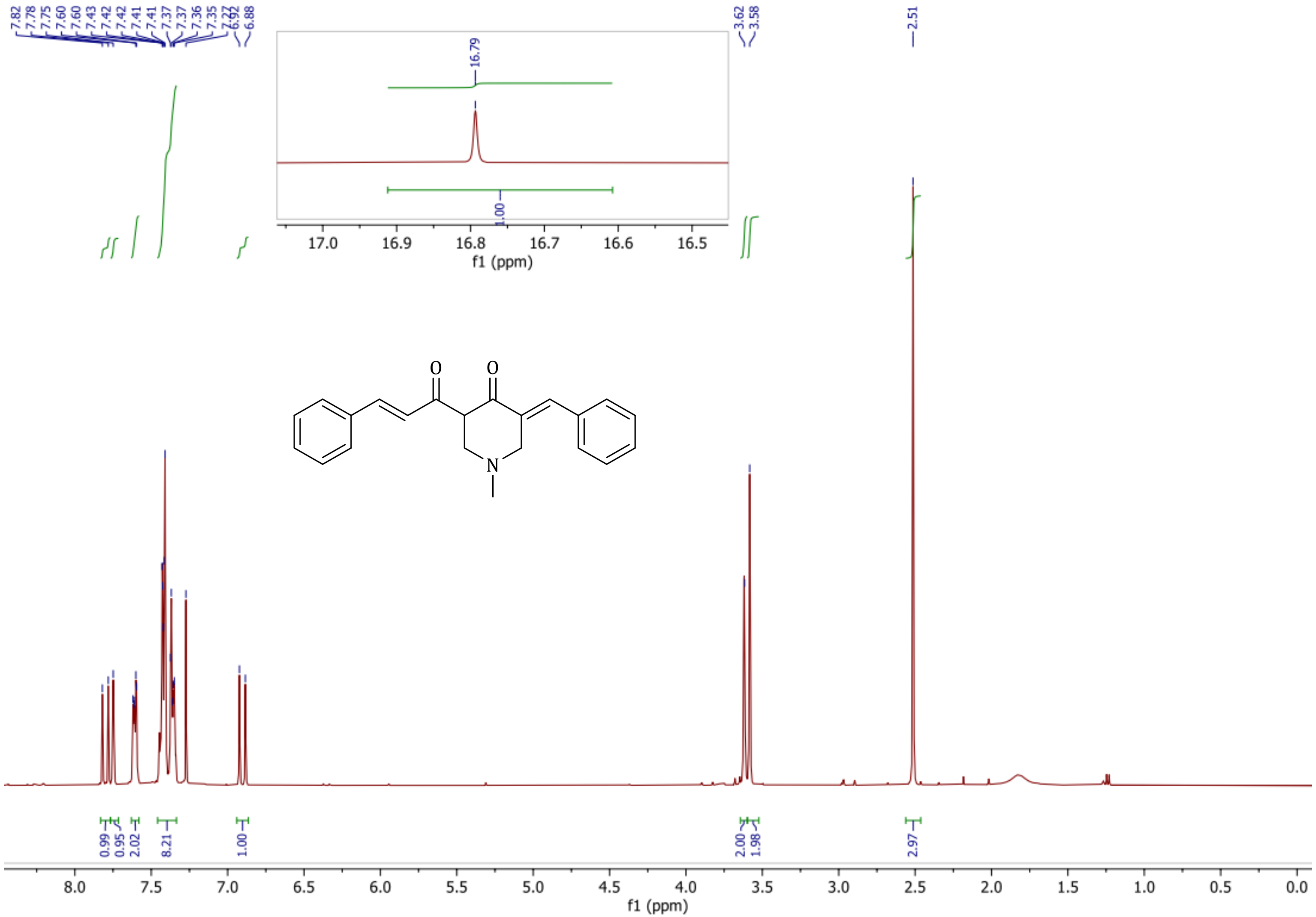

3-((*E*)-benzylidene)-5-cinnamoyl-1-methylpiperidin-4-one (**4**) ^{13}C NMR (100 MHz, $CDCl_3$)

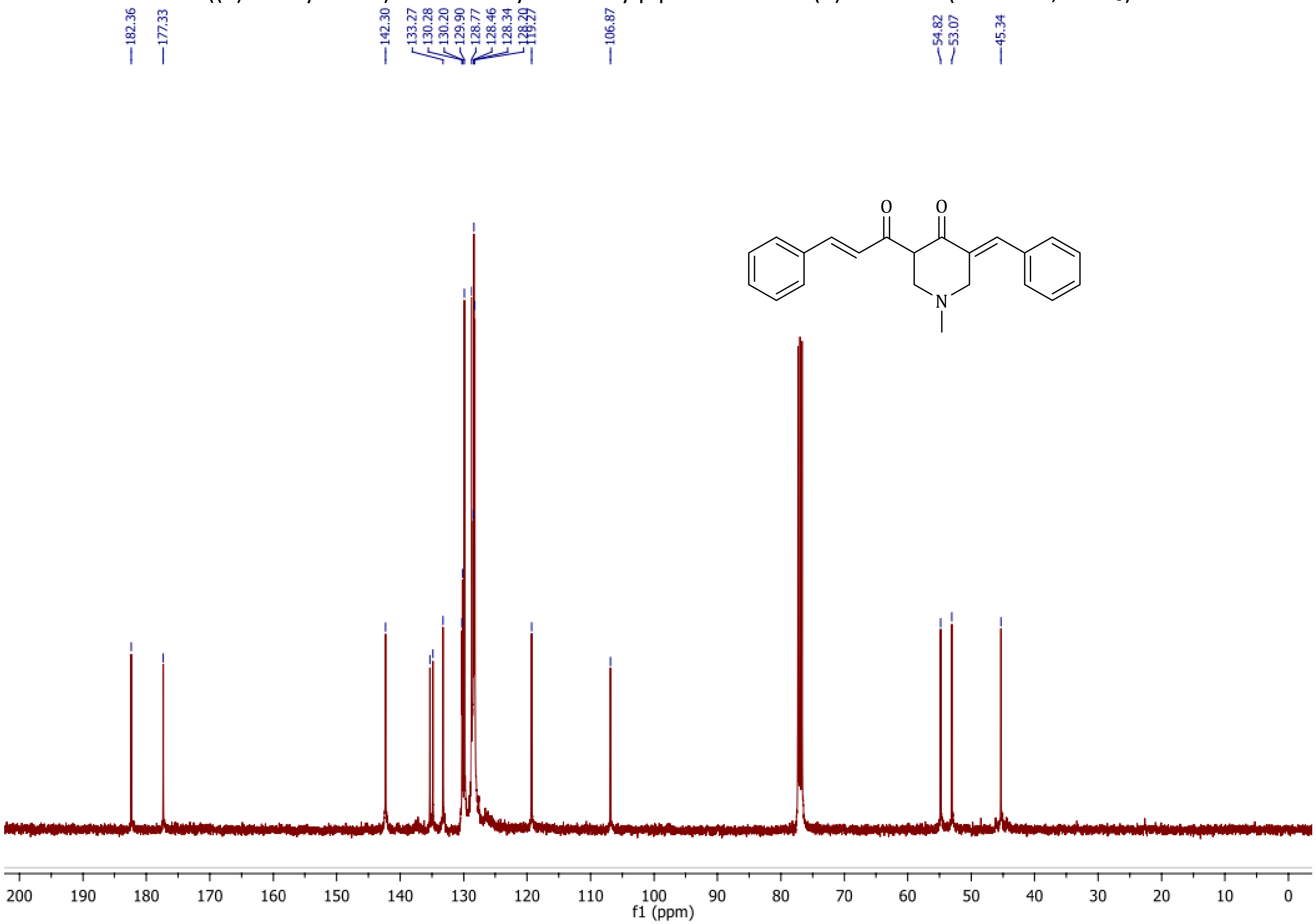

IR spectra of compound **4**; Attenuated Total Reflectance (ATR)

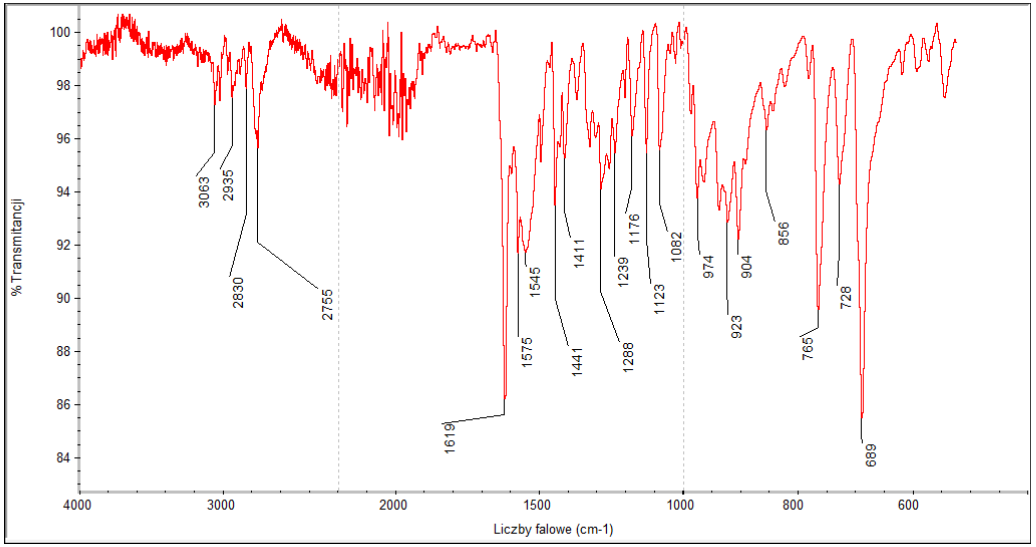

Compound **4** - HRMS (ESI): calcd. for: $C_{22}H_{22}NO_2$ $[M+H]^+$ 332.1645

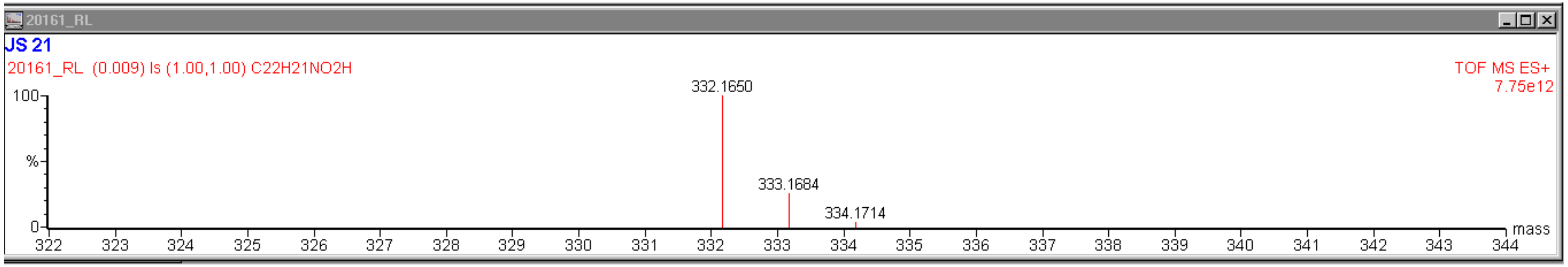

3-Cinnamoyl-5-((*E*)-4-hydroxy-3-methoxybenzylidene)-1-methylpiperidin-4-one (**5**) ^{1}H NMR (400 MHz, $CDCl_3$)

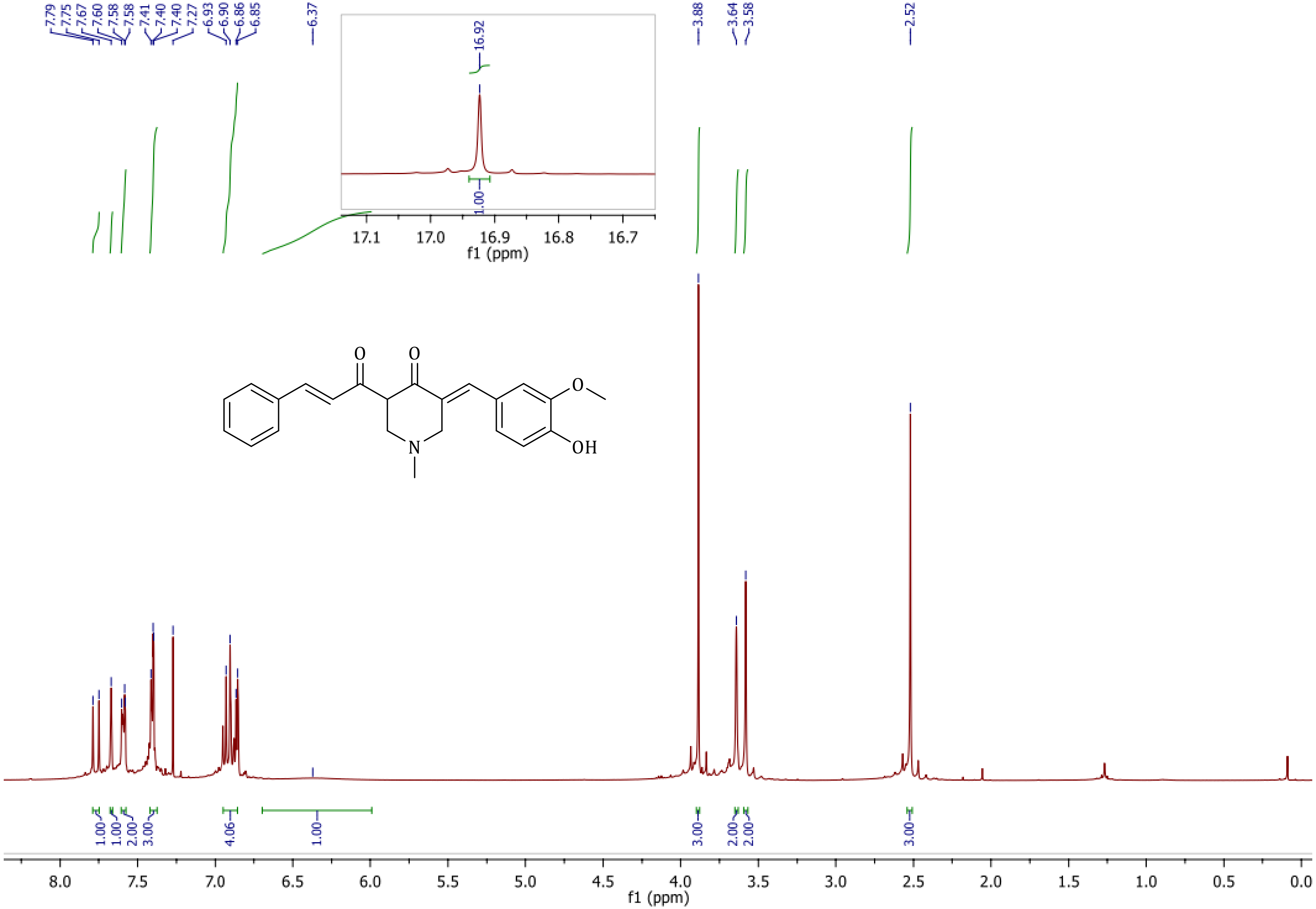

3-Cinnamoyl-5-((*E*)-4-hydroxy-3-methoxybenzylidene)-1-methylpiperidin-4-one (**5**) ^{13}C NMR (100 MHz, $CDCl_3$)

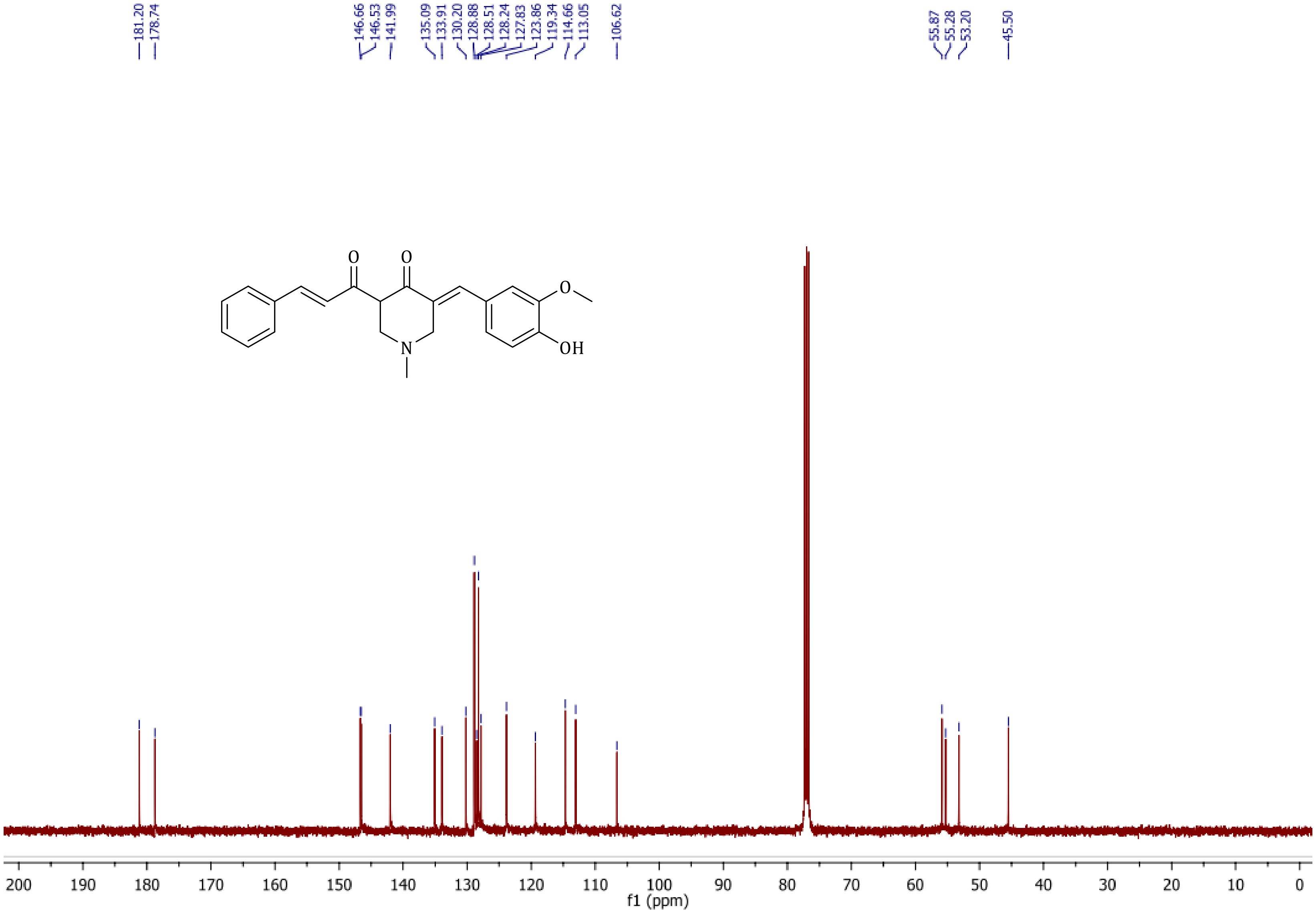

IR spectra of compound **5**; Attenuated Total Reflectance (ATR)

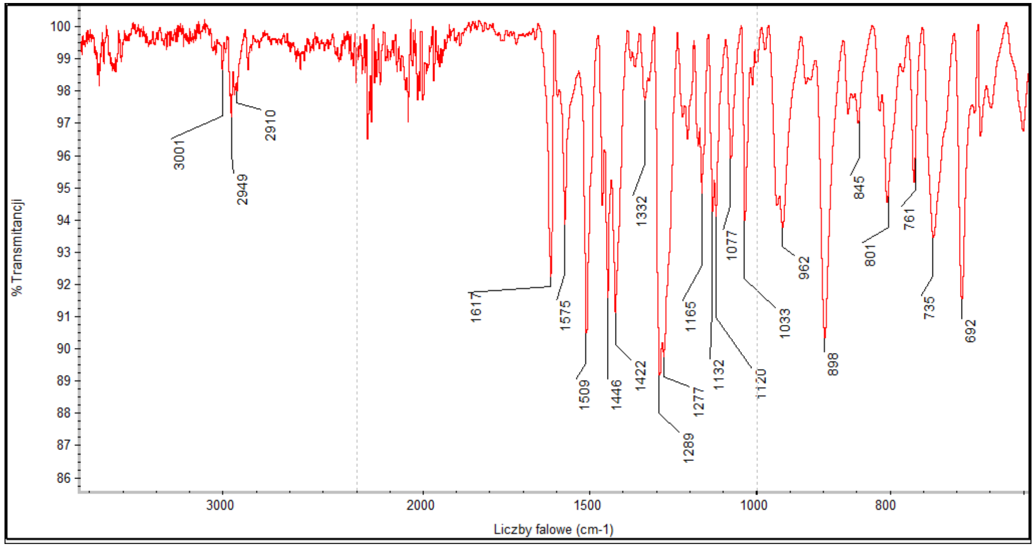

Compound **5**- HRMS (ESI): calcd. for: $C_{23}H_{24}NO_4$ $[M+H]^+$ 378.1700

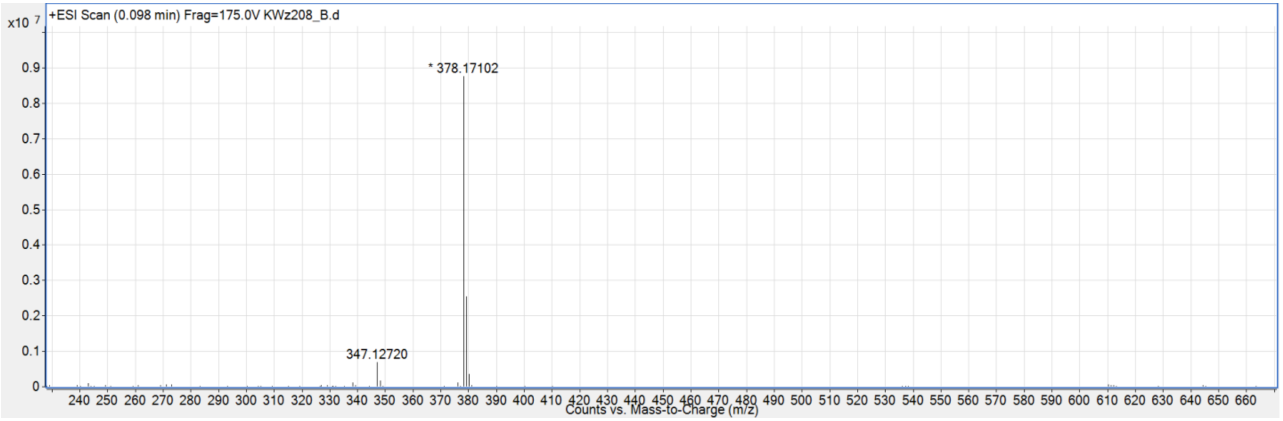

4-((*E*)-(5-Cinnamoyl-1-methyl-4-oxopiperidin-3-ylidene)methyl)-2-methoxyphenyl methyl carbonate (**6**) ^{1}H NMR (400 MHz, $CDCl_3$)

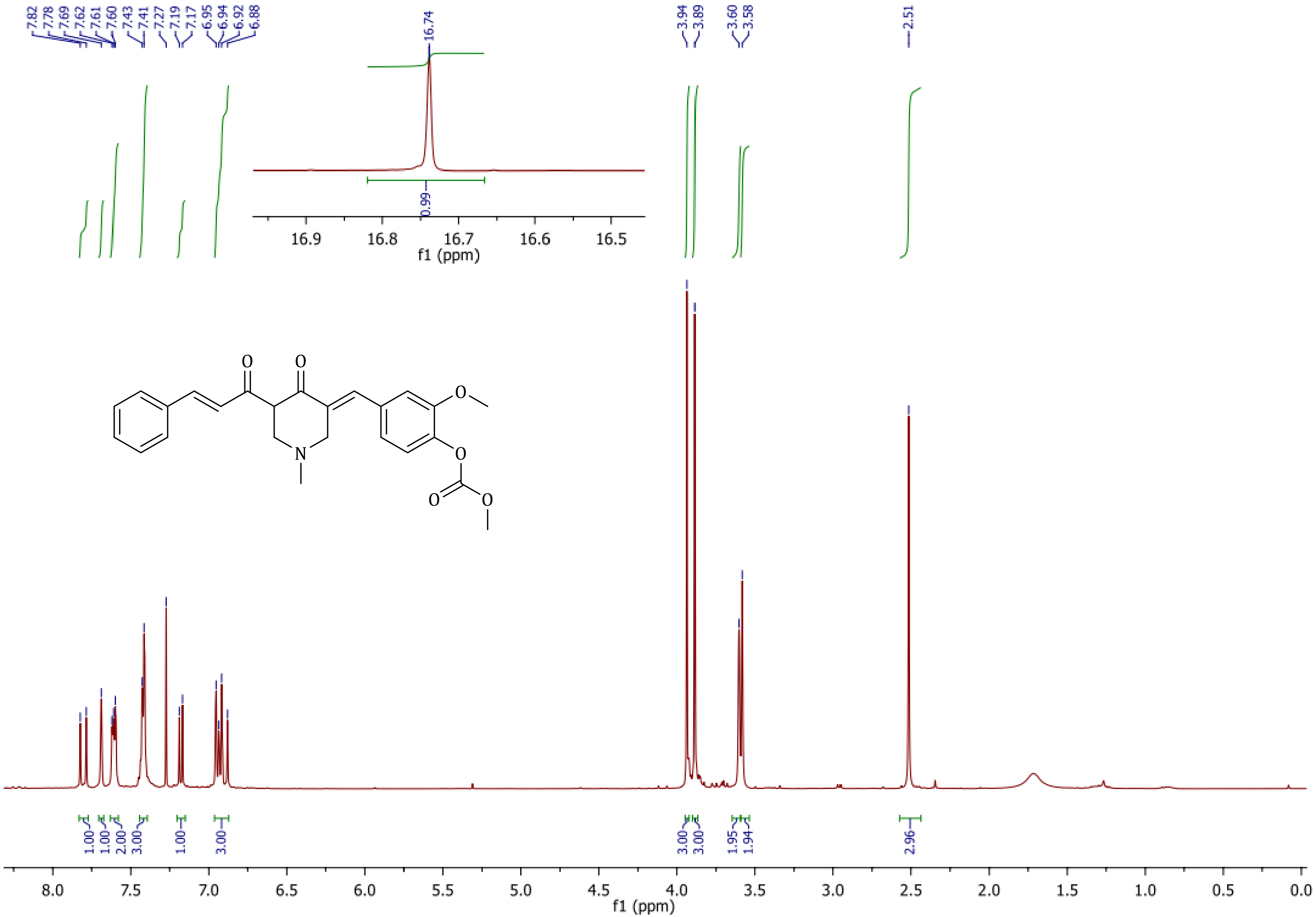

4-((*E*)-(5-Cinnamoyl-1-methyl-4-oxopiperidin-3-ylidene)methyl)-2-methoxyphenyl methyl carbonate (**6**) ^{13}C NMR (100 MHz, $CDCl_3$)

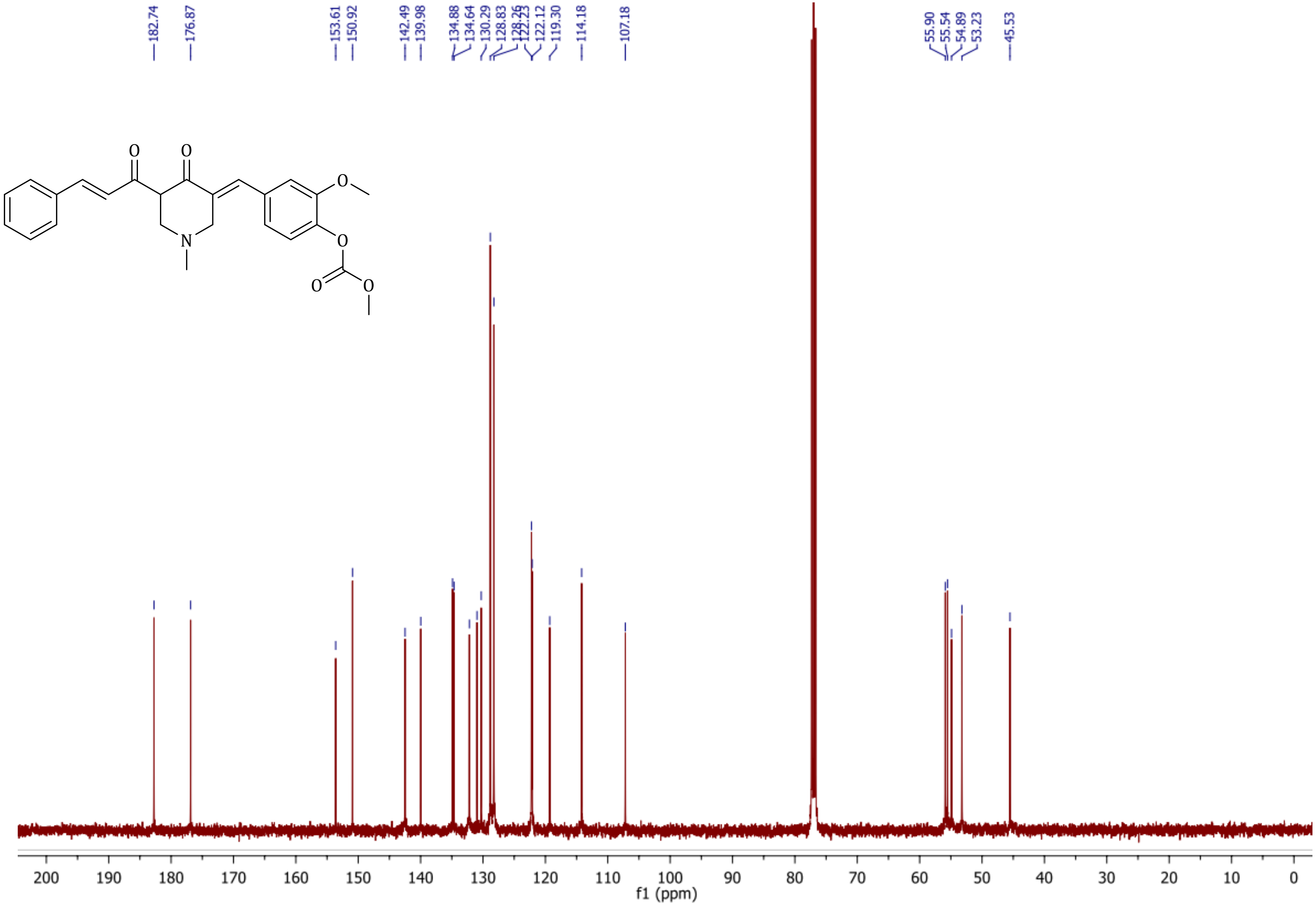

IR spectra of compound **6**; Attenuated Total Reflectance (ATR)

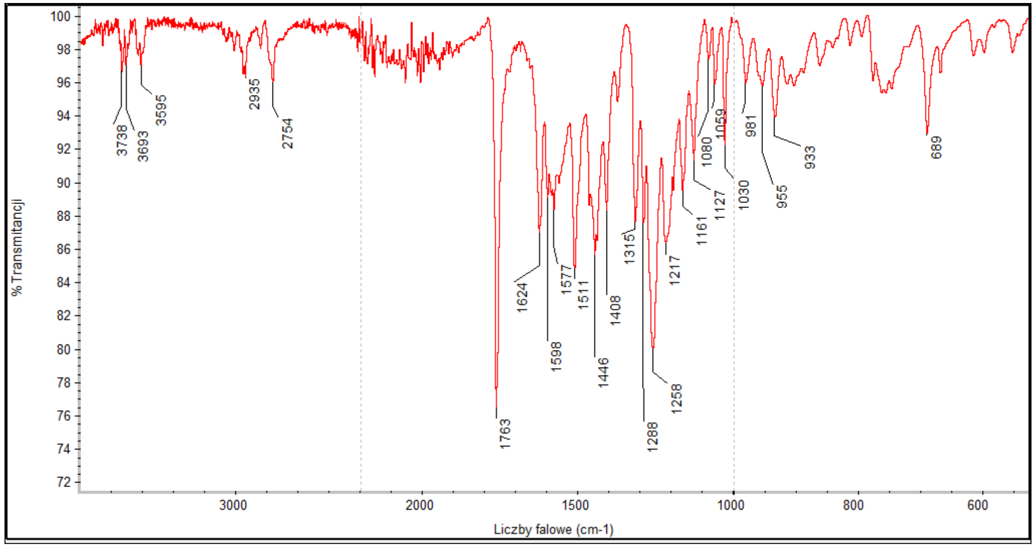

Compound **6** - HRMS (ESI): calcd. for: $C_{25}H_{26}NO_6$ $[M+H]^+$ 436.1755

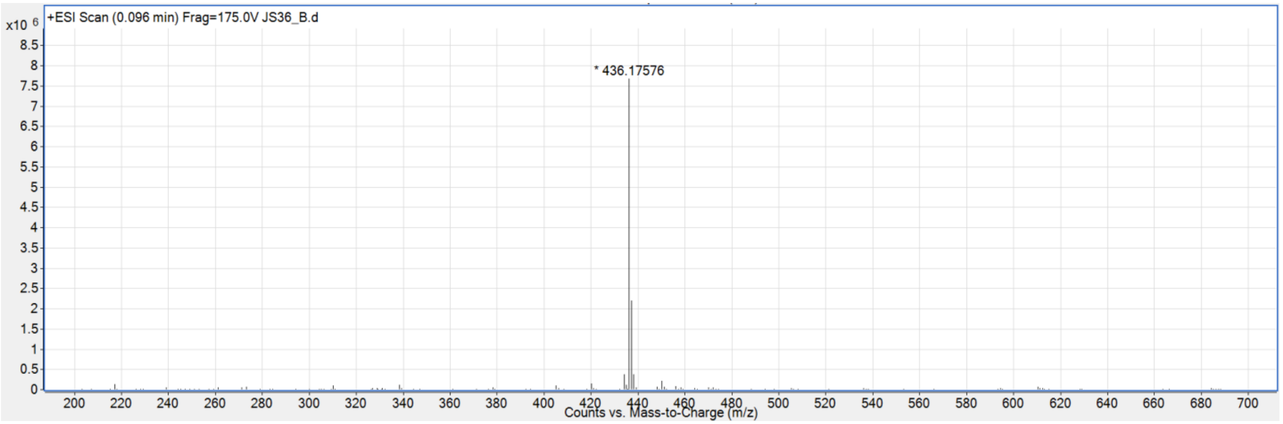

(*E*)-3-Benzylidene-5-((*E*)-3-(4-hydroxy-3-methoxyphenyl)acryloyl)-1-methylpiperidin-4-one (**7**) ^{1}H NMR (400 MHz, $CDCl_3$)

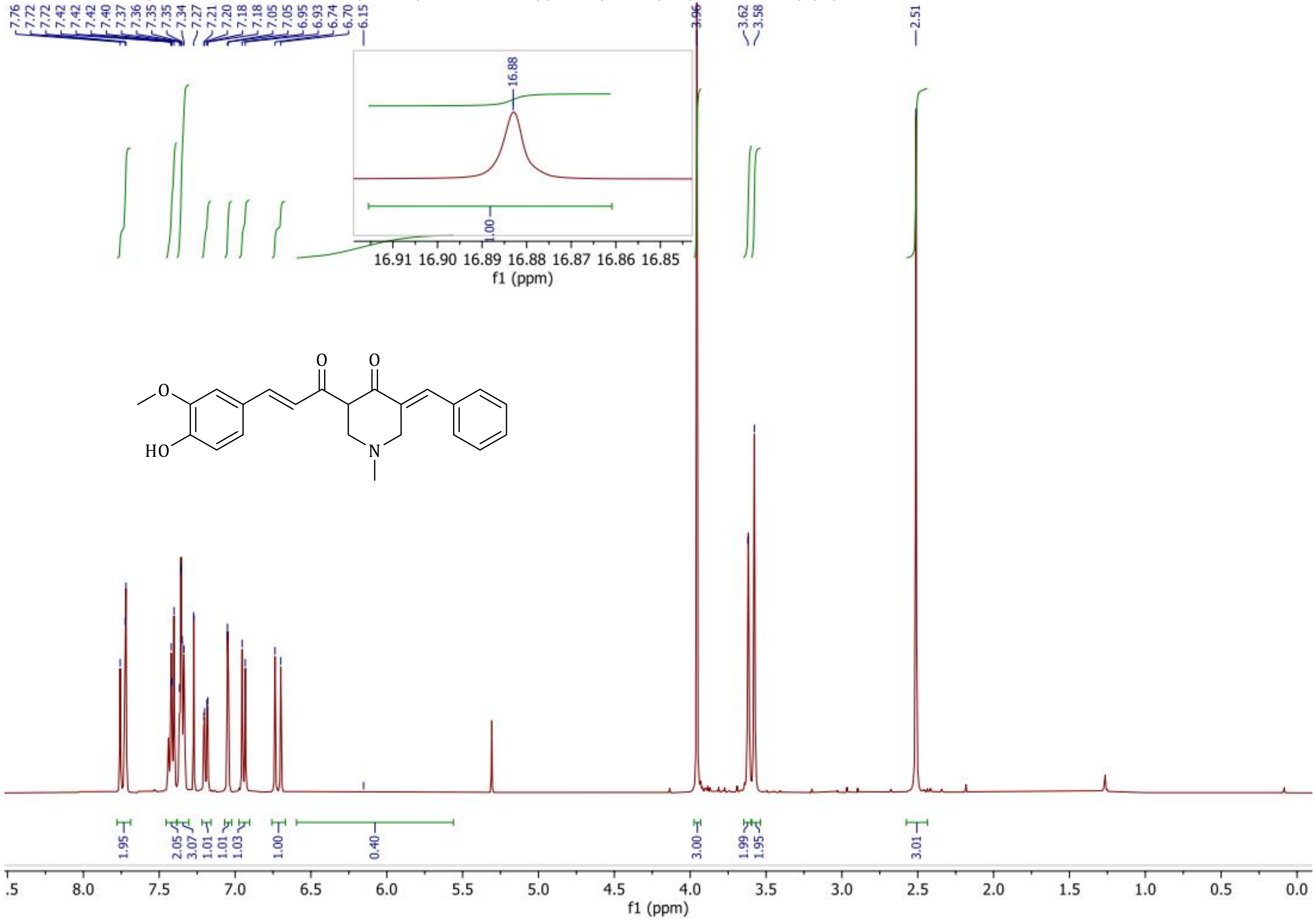

(*E*)-3-Benzylidene-5-((*E*)-3-(4-hydroxy-3-methoxyphenyl)acryloyl)-1-methylpiperidin-4-one (**7**) ^{13}C NMR (100 MHz, $CDCl_3$)

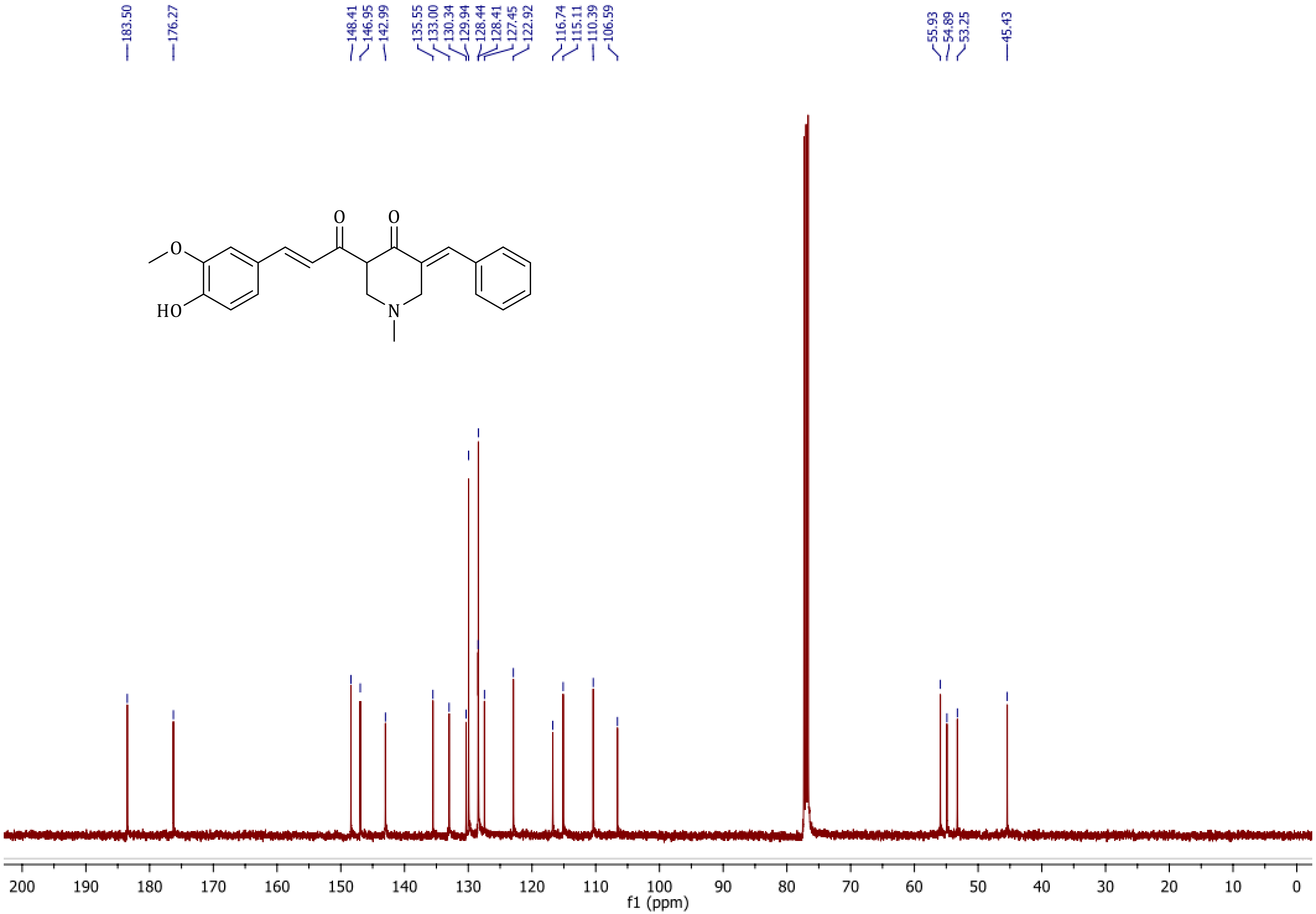

IR spectra of compound **7**; Attenuated Total Reflectance (ATR)

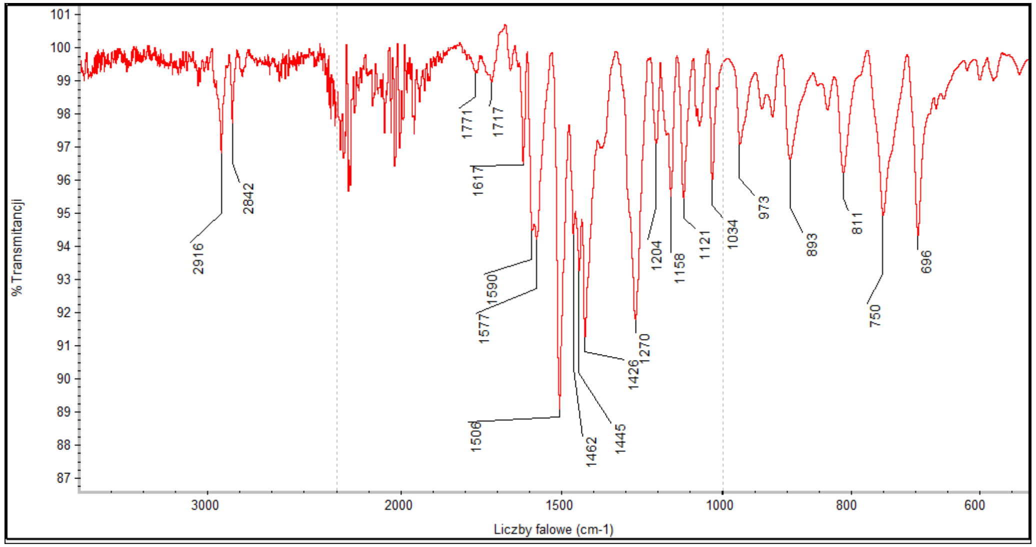

Compound **7** - HRMS (ESI): calcd. for: $C_{23}H_{24}NO_4$ $[M+H]^+$ 378.1700

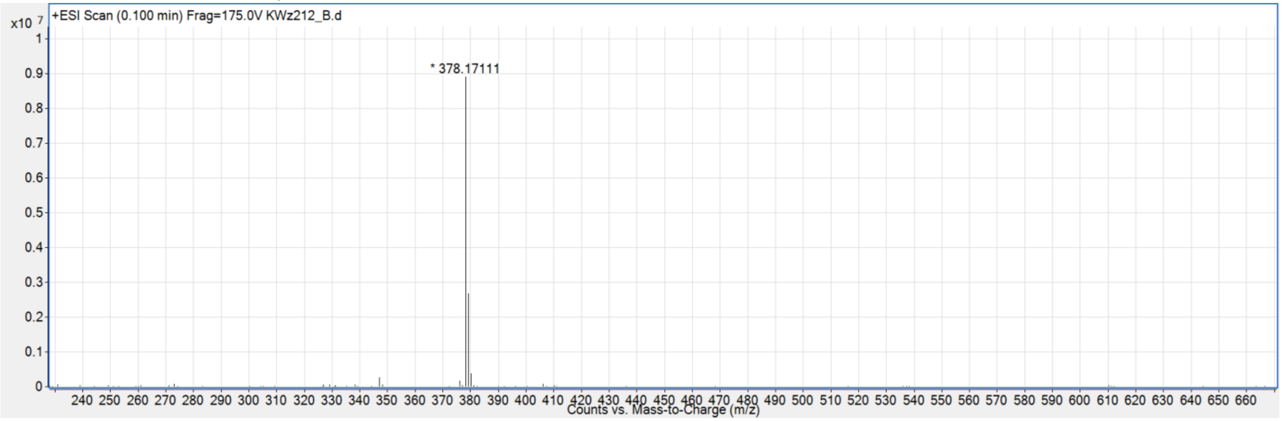

4-((*E*)-3-(5-((*E*)-Benzylidene)-1-methyl-4-oxopiperidin-3-yl)-3-oxoprop-1-en-1-yl)-2-methoxyphenyl methyl carbonate (**8**) ^{1}H NMR (400 MHz, $CDCl_3$)

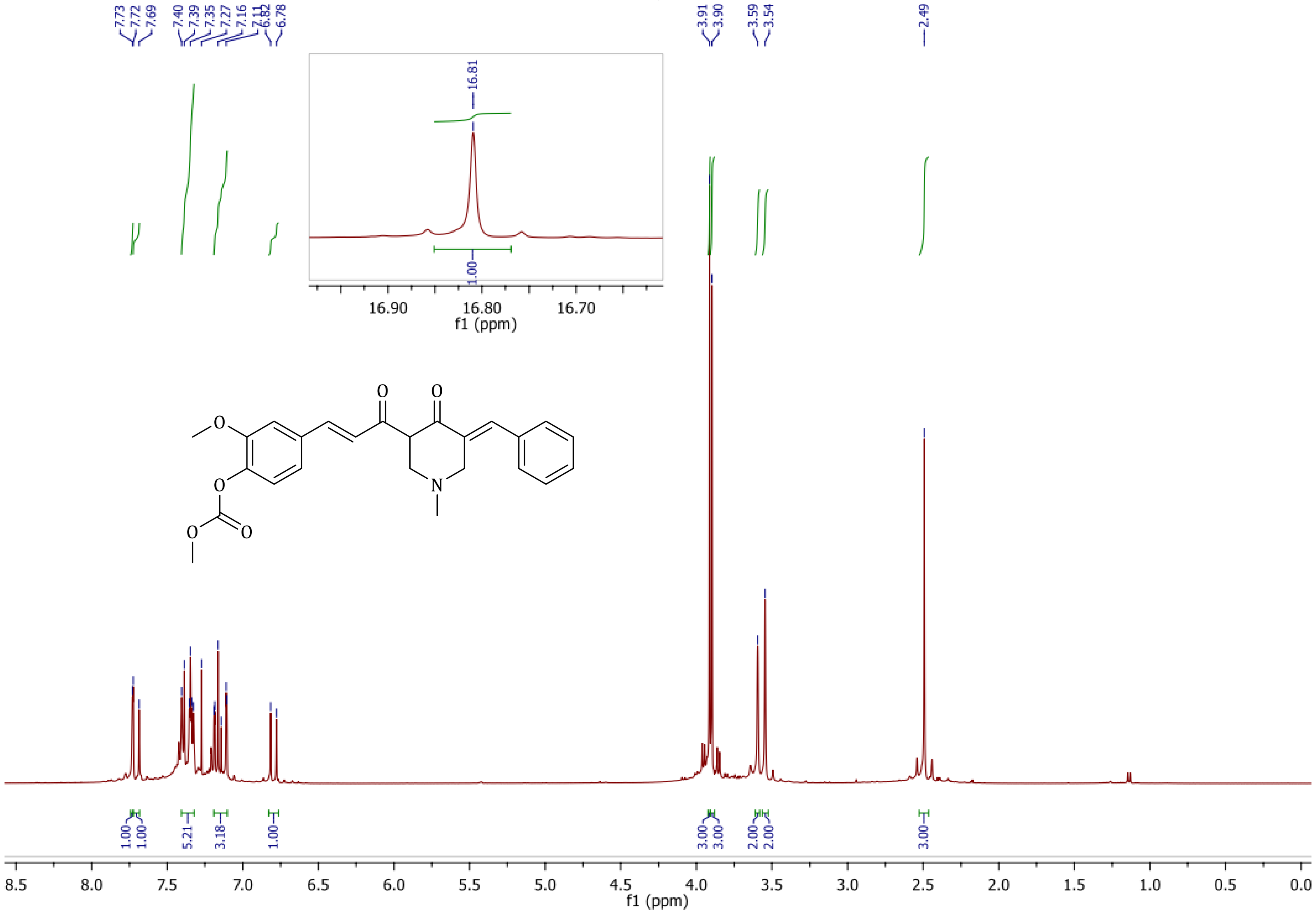

4-((*E*)-3-(5-((*E*)-Benzylidene)-1-methyl-4-oxopiperidin-3-yl)-3-oxoprop-1-en-1-yl)-2-methoxyphenyl methyl carbonate (**8**) ^{13}C NMR (100 MHz, $CDCl_3$)

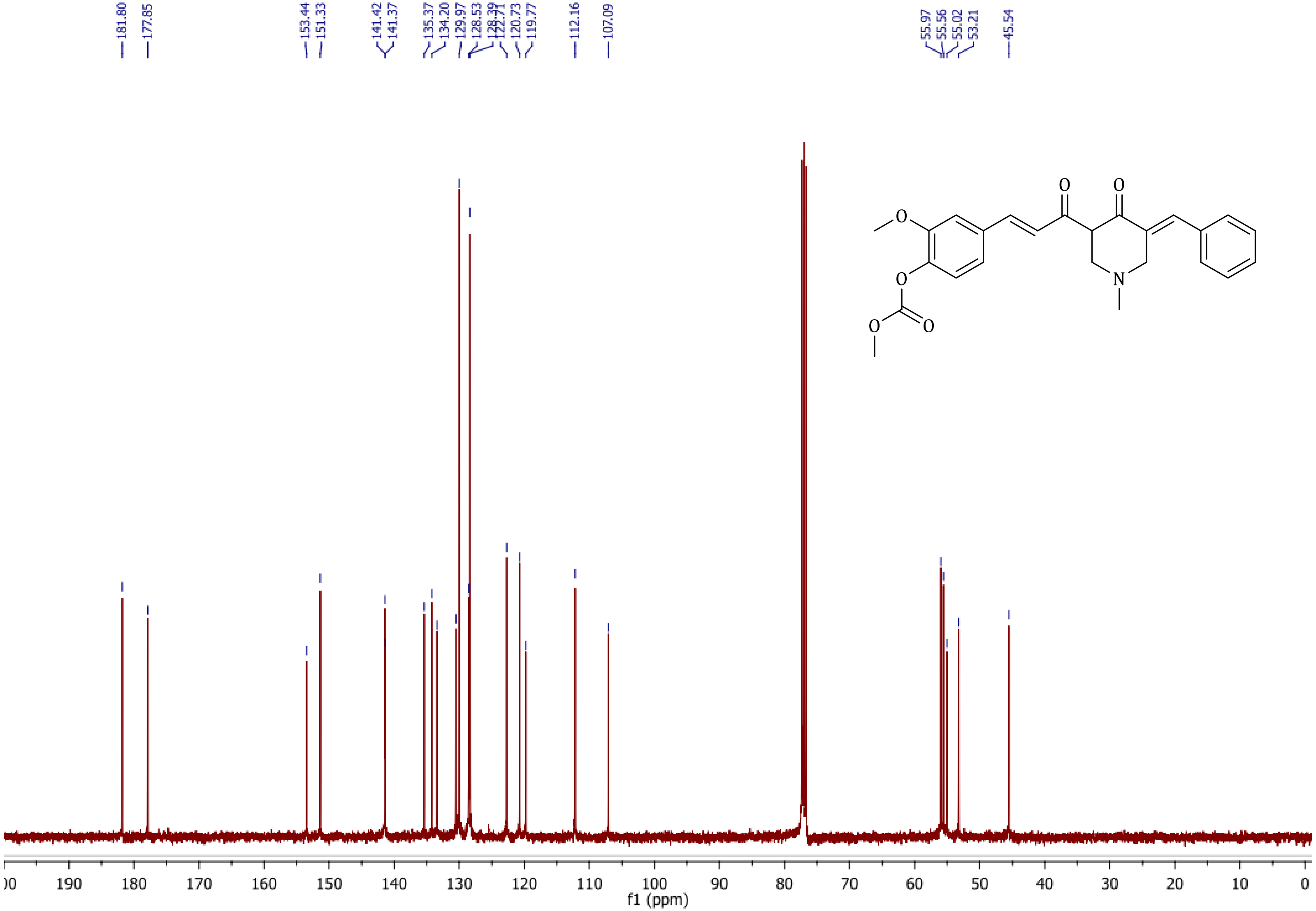

IR spectra of compound **8**; Attenuated Total Reflectance (ATR)

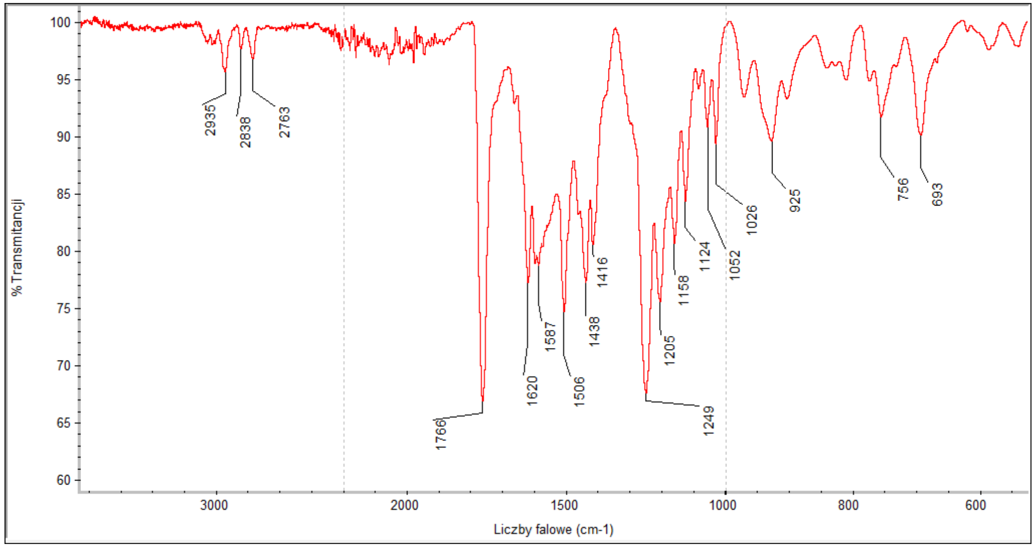

Compound **8** - HRMS (ESI): calcd. for: $C_{25}H_{26}NO_6$ $[M+H]^+$ 436.1755

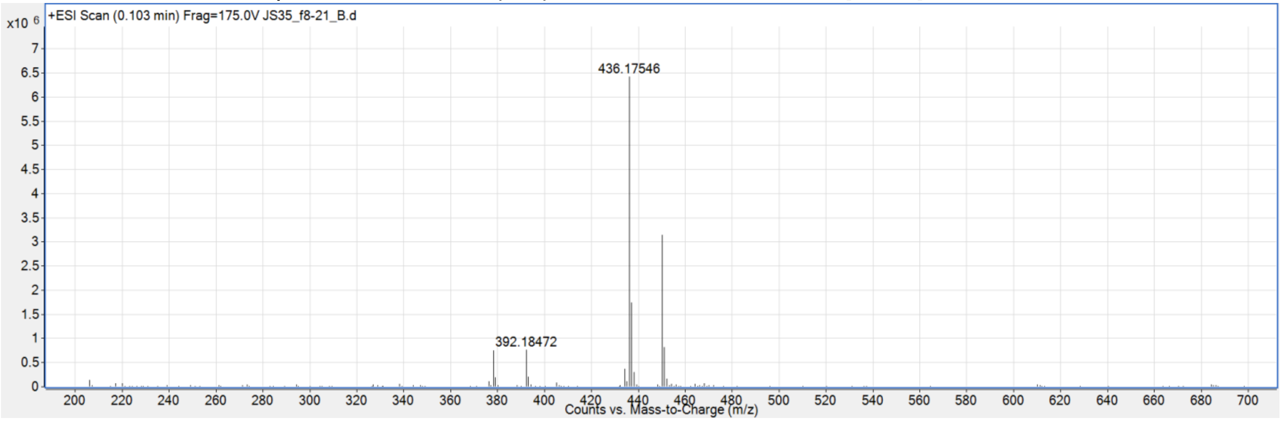

(3*E*,5*E*)-3,5-Bis(4-hydroksy-3-metoksyfenylo)-1-metylopiperyd-4-on (**9**) ^{1}H NMR (400 MHz, DMSO)

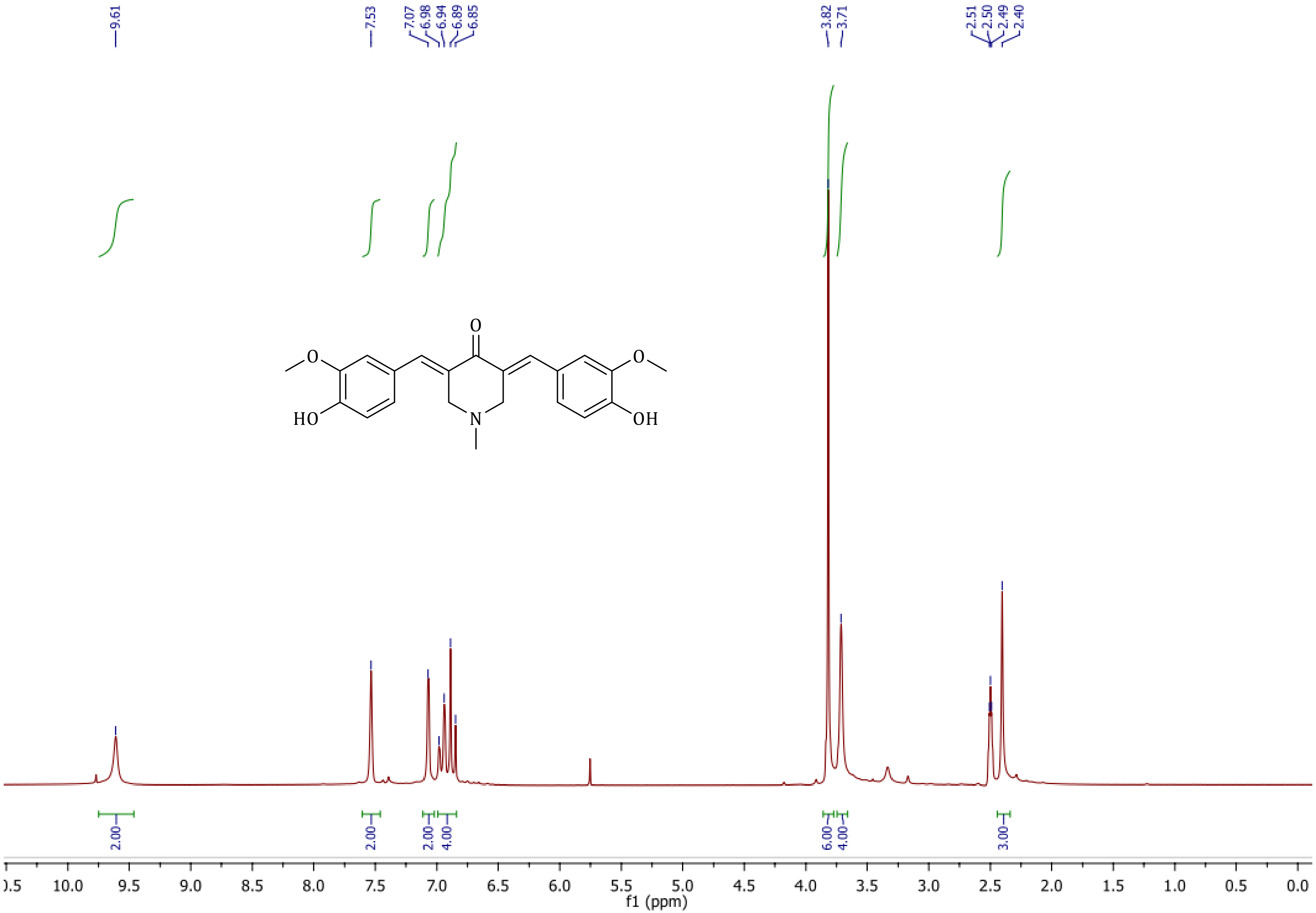

Dimethyl (((1*E*,1'*E*)-(1-methyl-4-oxopiperidine-3,5-diylidene)bis(methaneylylidene))bis(2-methoxy-4,1-phenylene)) bis(carbonate) (**10**) ^{1}H NMR (400 MHz, $CDCl_3$)

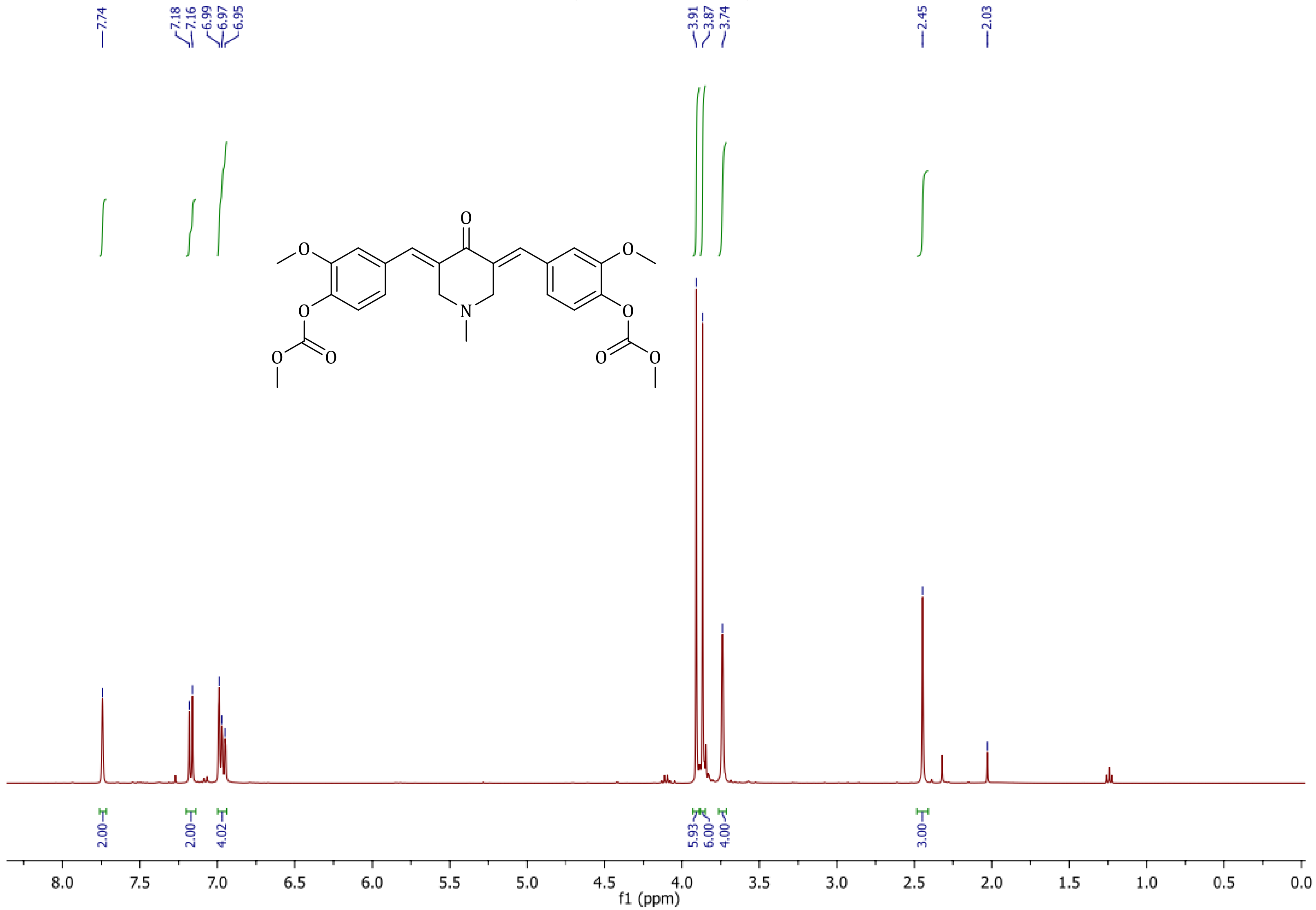

Dimethyl (((1*E*,1'*E*)-(1-methyl-4-oxopiperidine-3,5-diylidene)bis(methaneylylidene))bis(2-methoxy-4,1-phenylene)) bis(carbonate) (**10**) ^{13}C NMR (100 MHz, $CDCl_3$)

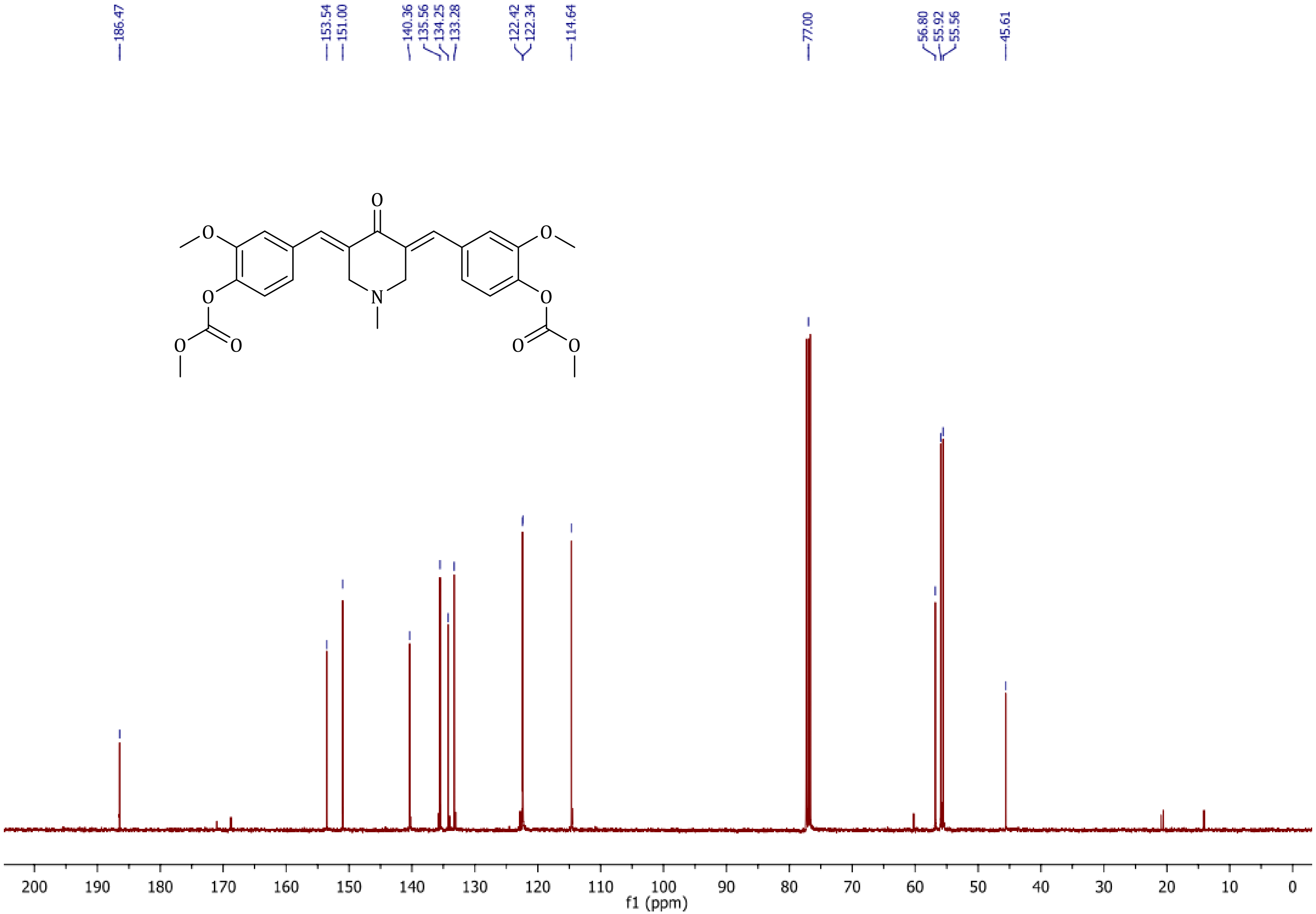

IR spectra of compound **10**; Attenuated Total Reflectance (ATR)

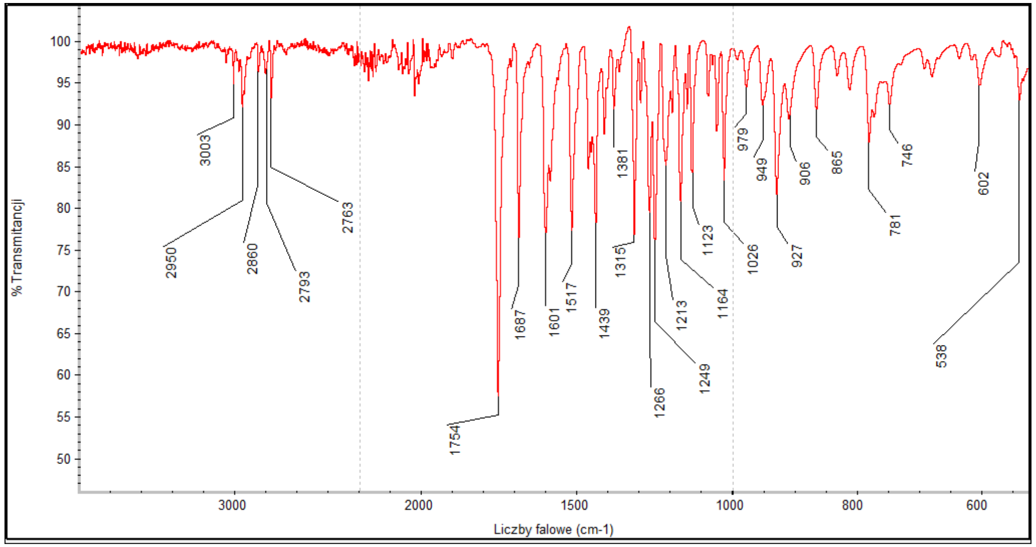

Compound **10** - HRMS (ESI): calcd. for: $C_{26}H_{28}NO_9$ $[M+H]^+$ 498.1759

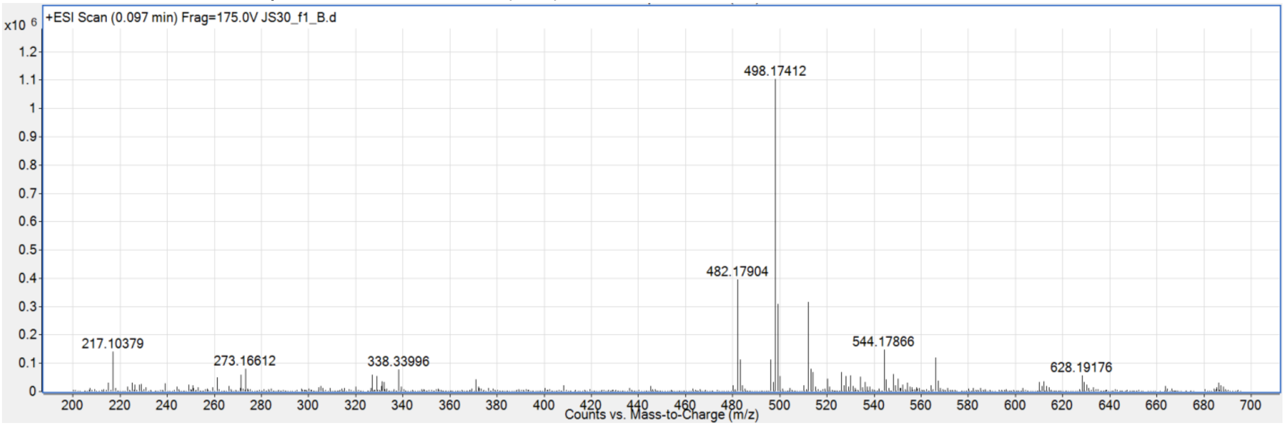

(3*E*,5*E*)-5-(4-Hydroksy-3-metoksybenzylideno)-3-[3-(4-hydroksy-3-metoksy)prop-2-enylo]-1-metylopiperyd-4-on (**11**) ^{1}H NMR (400 MHz, $CDCl_3$+CD3OD)

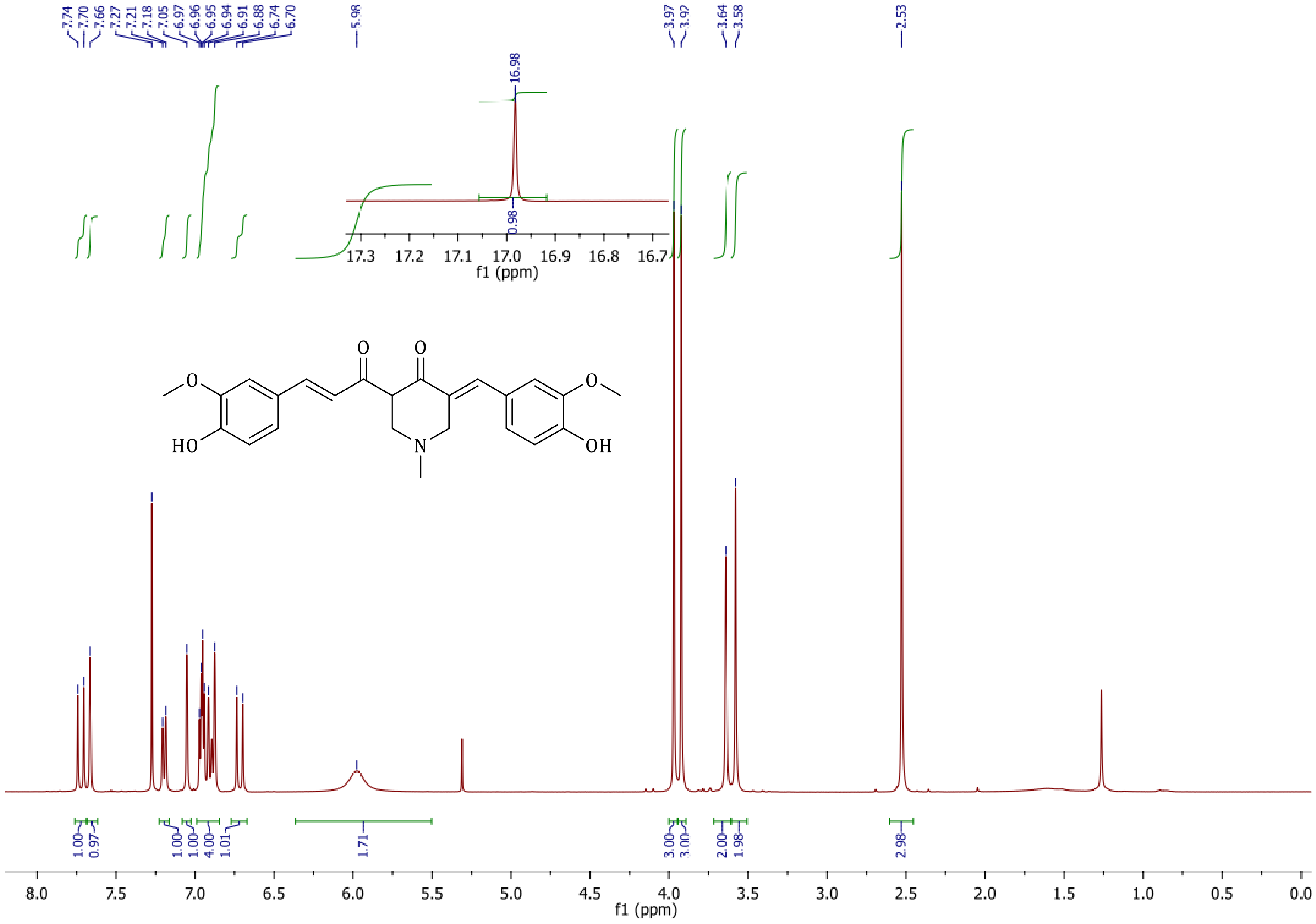

(3*E*,5*E*)-5-(4-Hydroksy-3-metoksybenzylideno)-3-[3-(4-hydroksy-3-metoksy)prop-2-enylo]-1-metylopiperyd-4-on (**11**) ^{13}C NMR (100 MHz, $CDCl_3$+CD3OD)

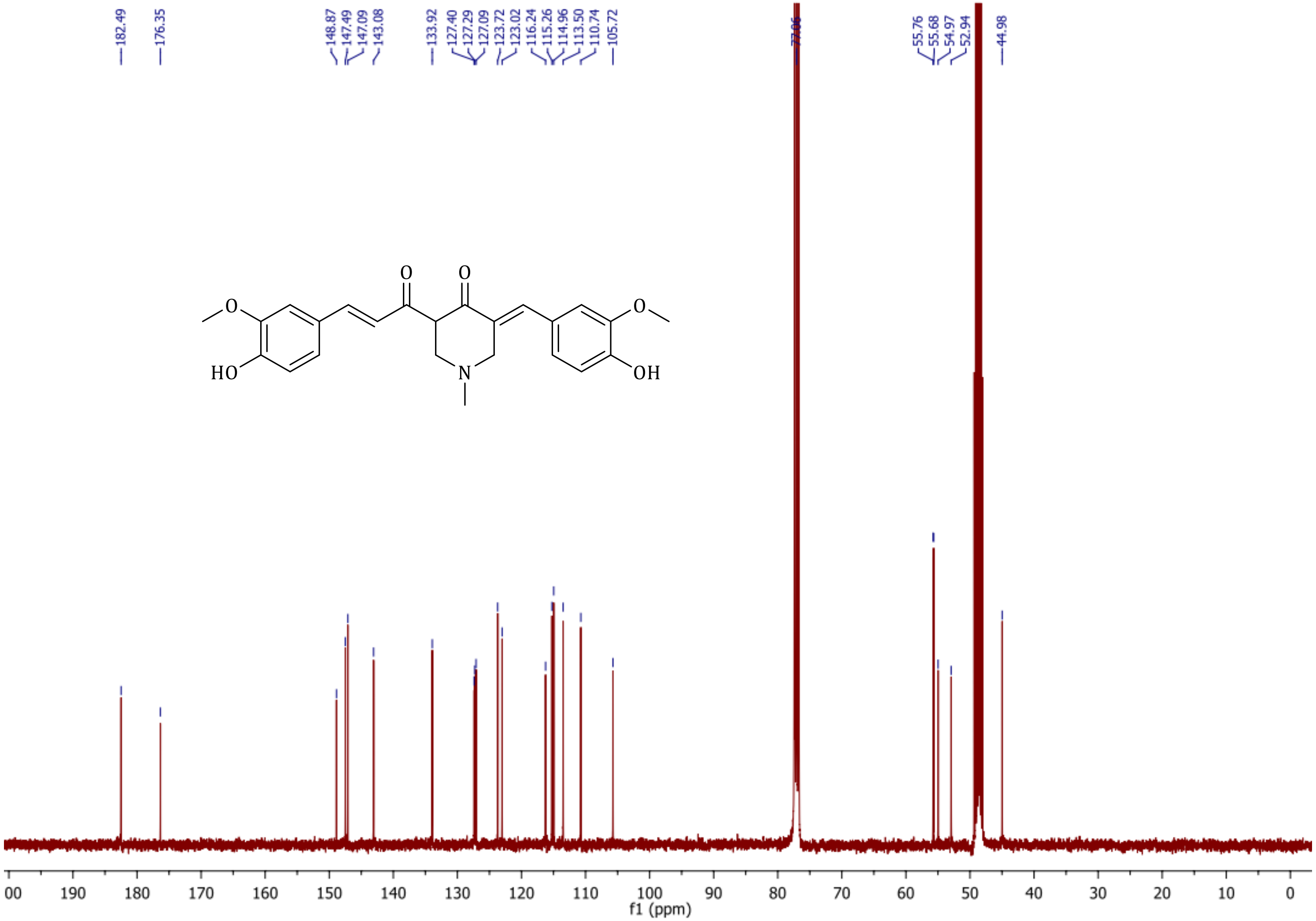

IR spectra of compound **11**; Attenuated Total Reflectance (ATR)

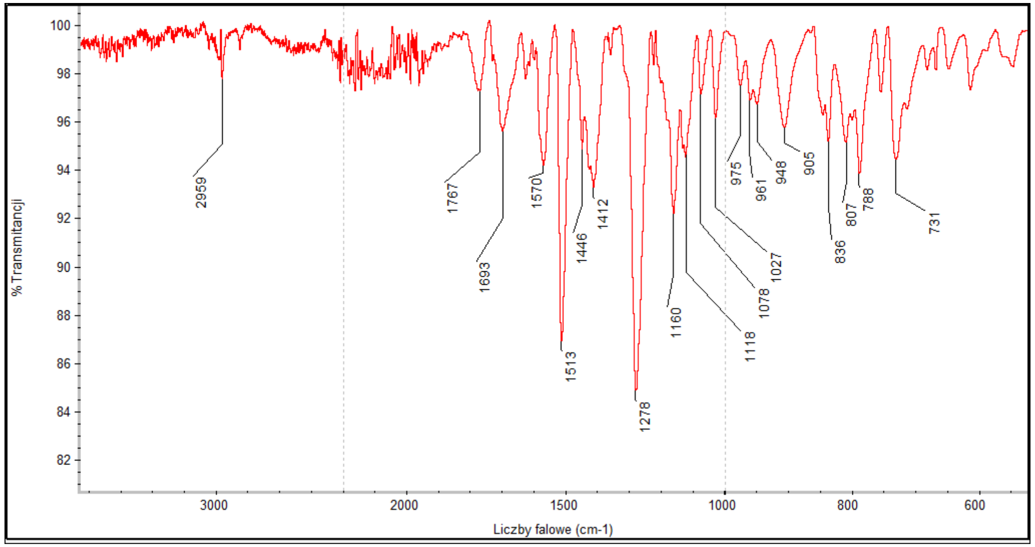

Compound **11** - HRMS (ESI): calcd. for: $C_{24}H_{26}NO_6$ $[M+H]^+$ 424.1755

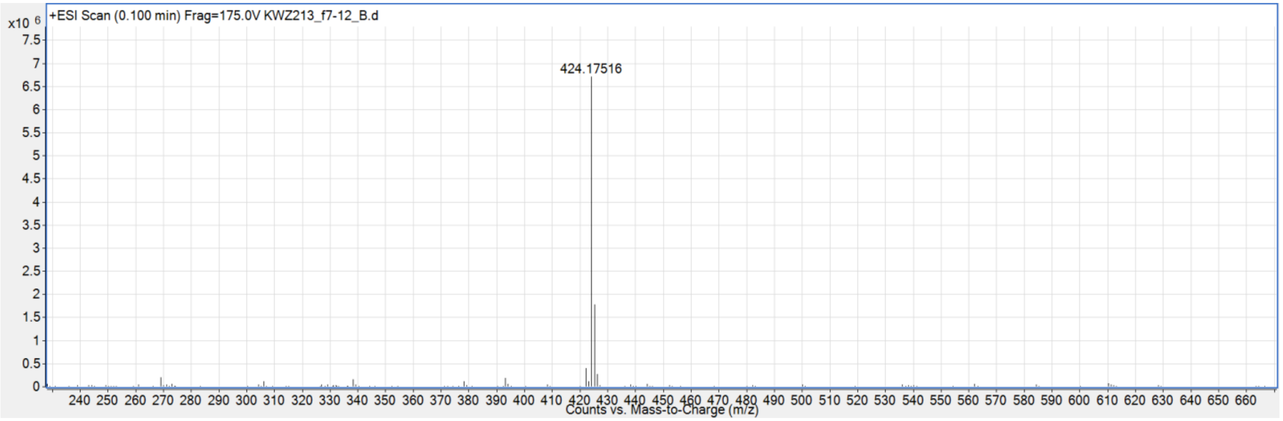

2-Methoxy-4-((*E*)-(5-((*E*)-3-(3-methoxy-4-((methoxycarbonyl)oxy)phenyl)acryloyl)-1-methyl-4-oxopiperidin-3-ylidene)methyl)phenyl methyl carbonate (**12**) ^{1}H NMR (400 MHz, $CDCl_3$)

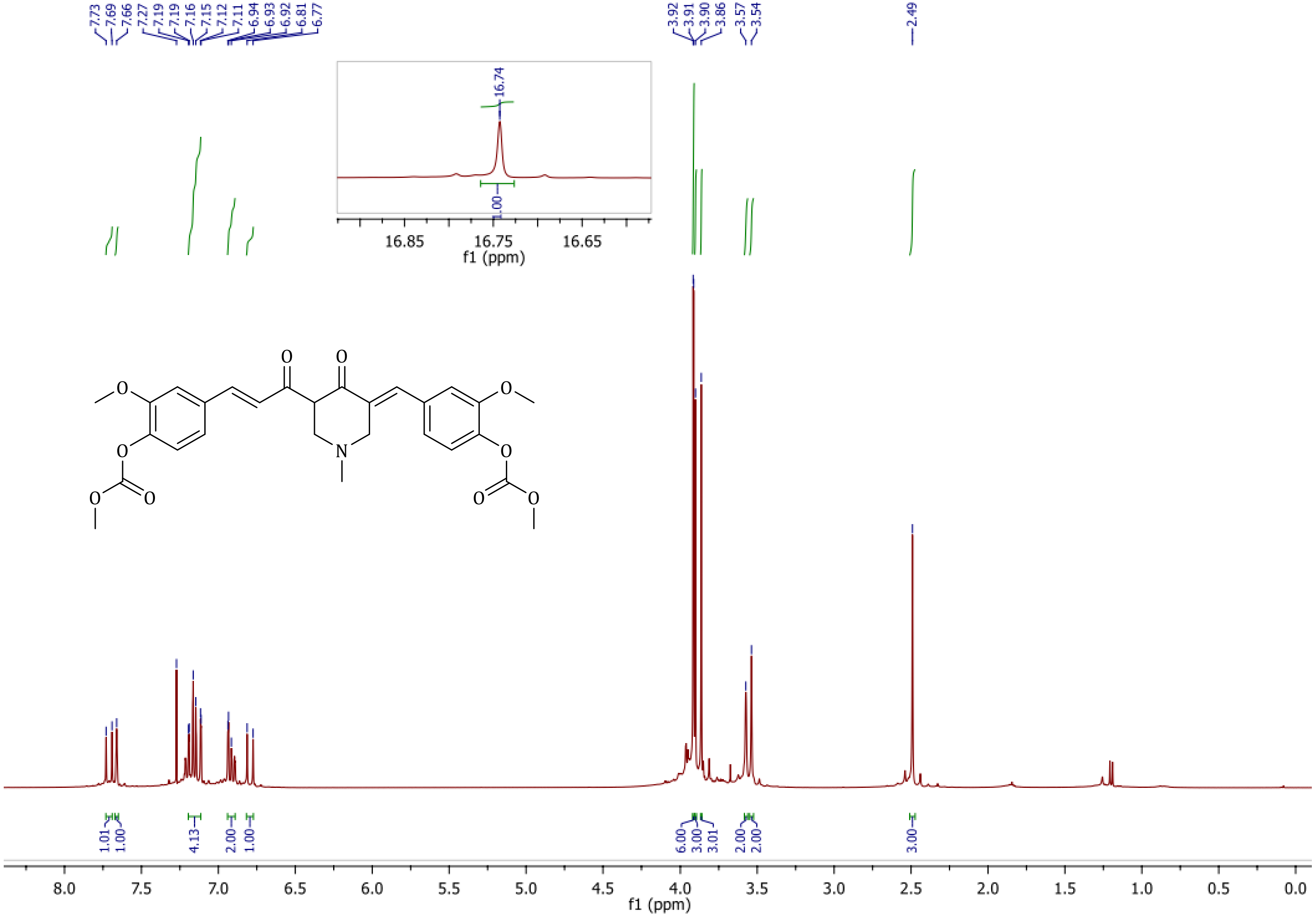

2-Methoxy-4-((*E*)-(5-((*E*)-3-(3-methoxy-4-((methoxycarbonyl)oxy)phenyl)acryloyl)-1-methyl-4-oxopiperidin-3-ylidene)methyl)phenyl methyl carbonate (**12**) ^{13}C NMR (100 MHz, $CDCl_3$)

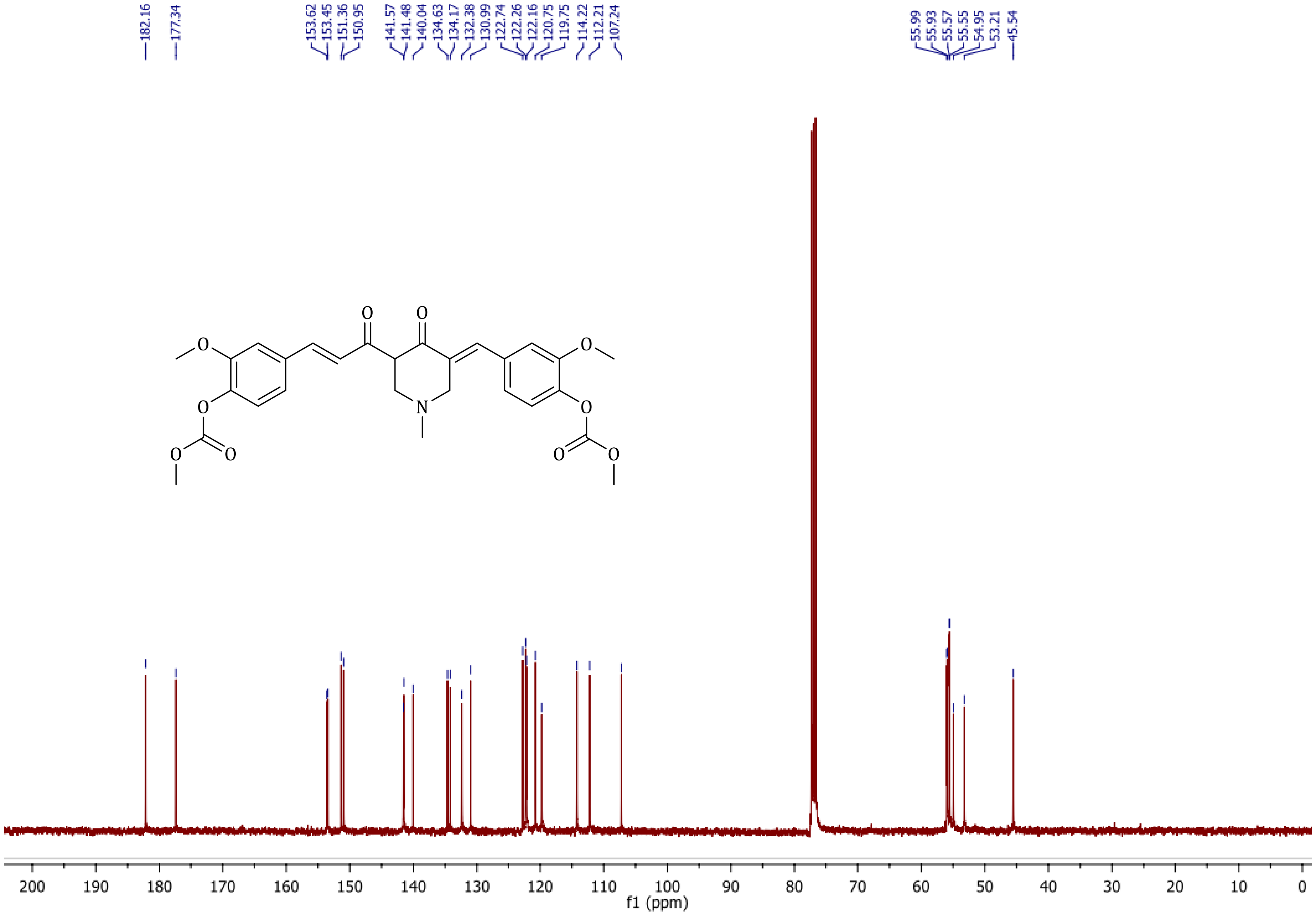

IR spectra of compound **12**; Attenuated Total Reflectance (ATR)

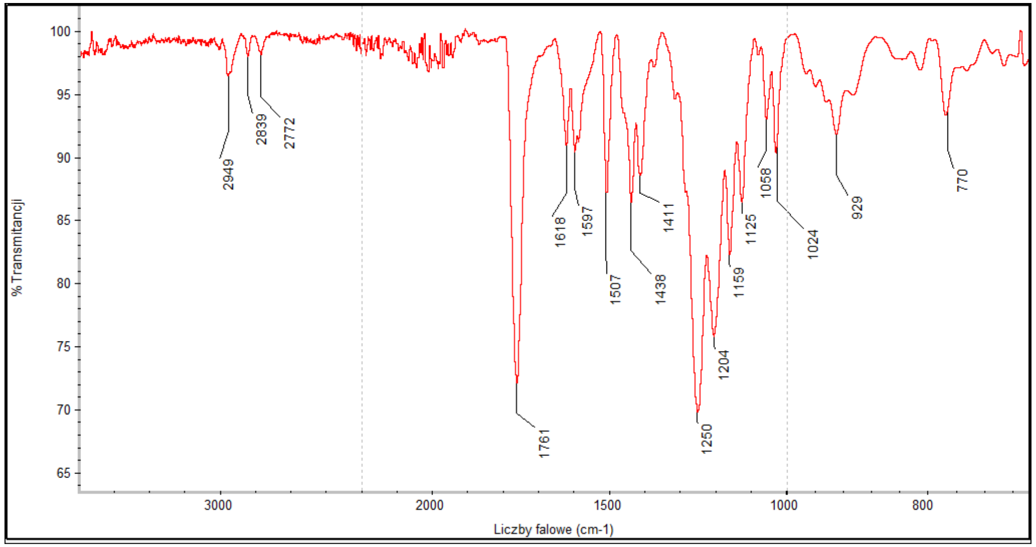

Compound **12** - HRMS (ESI): calcd. for: $C_{28}H_{30}NO_{10}$ $[M+H]^+$ 540.1864

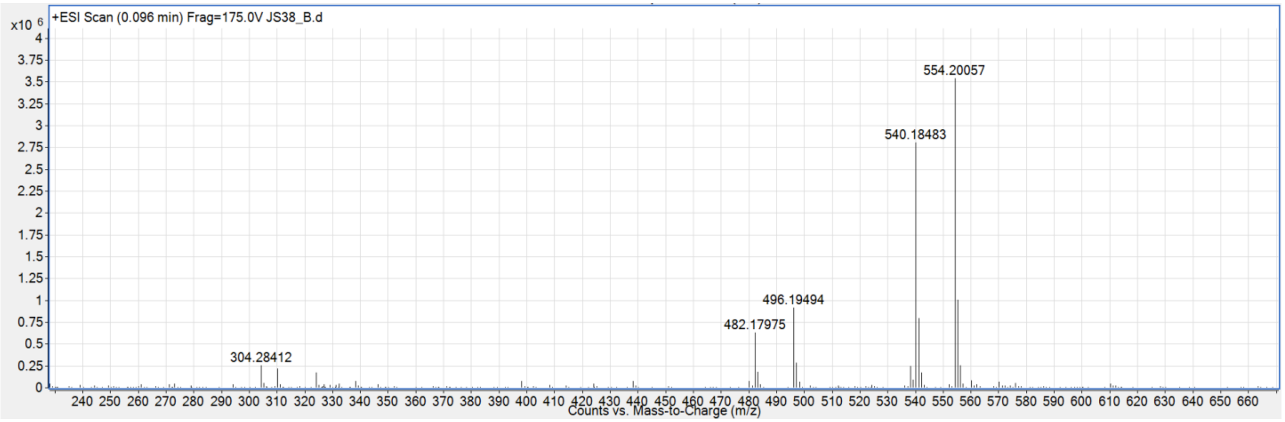

Supplement: Supplementary file 1 [file ijms-22-10368-s001.zip › ijms-1375127-supplementary.pdf]
